# The inflammatory cytokine TNFα cooperates with Ras in elevating metastasis and turns WT-Ras to a tumor-promoting entity in MCF-7 cells

**DOI:** 10.1186/1471-2407-14-158

**Published:** 2014-03-06

**Authors:** Tal Leibovich-Rivkin, Yulia Liubomirski, Tsipi Meshel, Anastasia Abashidze, Daphna Brisker, Hilla Solomon, Varda Rotter, Miguel Weil, Adit Ben-Baruch

**Affiliations:** 1 Department Cell Research and Immunology, George S. Wise Faculty of Life Sciences, Tel Aviv University, Tel Aviv 69978, Israel; 2 Department Molecular Cell Biology, Weizmann Institute of Science, Rehovot, Israel

**Keywords:** CXCL8, Interleukin 1β, p53, Ras, Tumor necrosis factor α

## Abstract

**Background:**

In the present study we determined the relative contribution of two processes to breast cancer progression: (1) Intrinsic events, such as activation of the Ras pathway and down-regulation of p53; (2) The inflammatory cytokines TNFα and IL-1β, shown in our published studies to be highly expressed in tumors of >80% of breast cancer patients with recurrent disease.

**Methods:**

Using MCF-7 human breast tumor cells originally expressing WT-Ras and WT-p53, we determined the impact of the above-mentioned elements and cooperativity between them on the expression of CXCL8 (ELISA, qRT-PCR), a member of a “cancer-related chemokine cluster” that we have previously identified. Then, we determined the mechanisms involved (Ras-binding-domain assays, Western blot, luciferase), and tested the impact of Ras + TNFα on angiogenicity (chorioallantoic membrane assays) and on tumor growth at the mammary fat pad of mice and on metastasis, in vivo.

**Results:**

Using Ras^G12V^ that recapitulates multiple stimulations induced by receptor tyrosine kinases, we found that Ras^G12V^ alone induced CXCL8 expression at the mRNA and protein levels, whereas down-regulation of p53 did not. TNFα and IL-1β potently induced CXCL8 expression and synergized with Ras^G12V^, together leading to amplified CXCL8 expression. Testing the impact of WT-Ras, which is the common form in breast cancer patients, we found that WT-Ras was not active in promoting CXCL8; however, TNFα has induced the activation of WT-Ras: joining these two elements has led to cooperative induction of CXCL8 expression, via the activation of MEK, NF-κB and AP-1. Importantly, TNFα has led to increased expression of WT-Ras in an active GTP-bound form, with properties similar to those of Ras^G12V^. Jointly, TNFα + Ras activities have given rise to increased angiogenesis and to elevated tumor cell dissemination to lymph nodes.

**Conclusions:**

TNFα cooperates with Ras in promoting the metastatic phenotype of MCF-7 breast tumor cells, and turns WT-Ras into a tumor-supporting entity. Thus, in breast cancer patients the cytokine may rescue the pro-cancerous potential of WT-Ras, and together these two elements may lead to a more aggressive disease. These findings have clinical relevance, suggesting that we need to consider new therapeutic regimens that inhibit Ras and TNFα, in breast cancer patients.

## Background

Recent studies have shown that sequential genetic/epigenetic alterations in intrinsic cellular components and the interactions between the tumor cells and their intimate microenvironment play major roles in the regulation of malignancy. The genetic/epigenetic modifications in intrinsic cellular components endow the tumor cells with the ability to circumvent normal regulatory processes. Well-defined alterations include the constitutive activation of Ras (e.g., Ras^G12V^) and the down-regulation of the tumor-suppressive activity of p53, which may be accompanied by oncogenic gain-of-function activity [1-4]. Interactions between tumor cells and their intimate microenvironment improve the abilities of those cells to propagate and metastasize. Here, major roles were recently identified to inflammatory cells and soluble inflammatory mediators that are present in the tumor microenvironment [4-8].

In a previously published study, we demonstrated the effects of these alterations and interactions on the ability of non-transformed cells to acquire a pro-malignancy phenotype, demonstrated by elevated expression of a “cancer-related chemokine cluster” [9]. This cluster included the highly angiogenic, malignancy-promoting chemokine CXCL8, as well as the tumor-promoting chemokine CCL2 [8,10-14]. We showed that the inflammatory cytokines tumor necrosis factor α (TNFα) and interleukin 1β (IL-1β), which have recently been suggested to promote malignancy [15-20], had a stronger effect on the malignancy phenotype of these cells than alterations in intrinsic cellular components did. We also found that Ras^G12V^ could not induce the chemokine cluster in the absence of cooperation with down-regulated p53 activities (e.g., down-regulation by shRNA) [9].

The relative roles played by intrinsic and microenvironmental factors may vary over the course of the malignancy process. Currently, information on the equilibrium between these two sets of factors in cancer and their ability to cooperate in dictating the angiogenic and malignancy phenotypes of tumor cells is relatively limited. In the present study, we used a well-defined cell system of human breast tumor cells (see below) to examine the interactions between these factors. We determined the effects of these factors on CXCL8 expression, using CXCL8 as a proxy for many pro-tumorigenic factors that may be induced in tumor cells. Then, we identified the joint effects of the intrinsic and inflammatory elements on angiogenesis, tumor growth and metastasis.

The inflammatory microenvironment was represented in our current study by TNFα and IL-1β. These cytokines are extensively expressed in the tumor cells of more than 80% of breast cancer patients with relapsed disease [21] and they have recently been identified as tumor-promoting entities (e.g., [15-26]). While having cytotoxic effects when acutely administered to tumors, the chronic presence of TNFα in breast tumor sites leads to increased tumor aggressiveness; IL-1β up-regulates processes that contribute to higher angiogenesis, tumor growth and progression in breast cancer (e.g., [21-26]). In parallel, we examined the Ras and p53 pathways. Ras has been shown to be hyper-activated in breast cancer patients due to excessive stimulation of receptor tyrosine kinases (RTKs), such as ErbB2, which is amplified in approximately 25% of the patients. Also, in about 25% of breast cancer patients, p53 is down-regulated [1,3,27-30]. Supporting our choice of TNFα and IL-1β, and of Ras and p53, are studies suggesting that these elements may be involved in the regulation of inflammatory chemokines in cancer ([21,31-34] and [35-39]).

In this study, we demonstrated that Ras^G12V^, which is the form of Ras that recapitulates the activation of Ras by multiple RTKs (as is the case in breast cancer), induced the release of CXCL8 and CCL2 from MCF-7 human breast tumor cells, without any need to cooperate with the down-regulation of p53. Moreover, in these cells TNFα and IL-1β cooperated with Ras^G12V^ to promote the expression of CXCL8 at the mRNA and protein levels. In parallel, we found that wild-type Ras (WT-Ras) has cooperated with TNFα, and these two elements together gave rise to the amplified expression and release of CXCL8 by the tumor cells. Also, signals delivered by TNFα increased the overall levels of the activated, GTP-bound form of WT-Ras, which then induced the up-regulation of CXCL8 expression through MEK, NF-κB and AP-1. Moreover, the joint activities of TNFα and activated Ras led to cooperative induction of angiogenesis and to increased dissemination of tumor cells to lymph nodes (LN).

The results obtained in our study propose that interactions between inflammatory factors and oncogenic pathways aggravate disease course in breast cancer, and are supported by several recent findings in the field [40,41]. If generalized through investigation in other suitable breast tumor systems, such mechanisms imply that in breast cancer patients whose tumors contain high levels of the inflammatory cytokine TNFα and whose cancer cells generally do not carry mutations in Ras, TNFα may activate WT-Ras towards a pro-cancerous phenotype that leads to devastating tumor-promoting outcomes. These results may have important clinical implications as they suggest that the use of inhibitors of mutated and thus hyper-activated Ras (such inhibitors are now in clinical trials, [2]) as well as inhibitors of TNFα (currently in use for the clinical treatment of autoimmune diseases [6]) may be considered in patients whose tumor cells do not carry any intrinsic Ras mutation, but do express high levels of TNFα, as is often the case in breast cancer and possibly in other malignancies as well.

## Methods

### Cells, vectors and transfections

The study was performed with MCF-7 cells, which are human luminal breast tumor cells that (1) Express WT-Ras [29,30]; (2) Express WT-p53 [30,42]; (3) Respond to TNFα and to IL-1β [21,32,43]. This cell line has provided the unique setup required for our study, as also described in the “Results” section. The cells were kindly given to us by Prof. Kaye (Weizmann Institute of Science, Rehovot, Israel) and were maintained in growth media containing DMEM supplemented by 10% fetal calf serum (FCS), 2 mM L-glutamine, 100 Units/ml penicillin, 100 μg/ml streptomycin and 250 ng/ml amphotericin (all from Biological Industries, Beit Haemek, Israel). The cells were authenticated on the basis of published characteristics of MCF-7 cells ([44] and reviewed in [45]) by verifying that they express an active estrogen receptor α, respond to estrogen, express low expression of ErbB2, form tumors upon supplementation of estrogen and matrigel and have low metastatic potential. In line with published reports on TNFα-induced cytolysis of MCF-7 cells, TNFα has induced cytolysis in ~15-30% of Ras-expressing cells.

MCF-7 cells were stably transfected by electroporation (using MP-100 MicroPorator, Digital Bio, Seoul, Korea; Transfection was performed according to manufacturer’s instructions) to express a well-recognized shRNA to p53 (on p-super-retro; Kindly provided by Prof. Agami, Netherlands Cancer Institute, Amsterdam, Netherlands) or the control vector. Following selection with 6 μg/ml puromycin (A.G. Scientific, San Diego, CA), the cell population was used as a whole in order to prevent bias towards specific cell clones, and p53 down-regulation was verified by Western blot (WB) (see “Results”). In parallel, MCF-7 cells were transiently transfected by electroporation (as described above) with GFP-H-Ras^G12V^ (=Ras^G12V^) or by control GFP-expressing vector (pEGFP-N3). The whole population of transfected cells was used, and Ras over-expression was verified by GFP expression (see “Results”). The activation of Ras^G12V^ was validated by Ras-binding-domain assays (see “Results”) and by elevated Erk phosphorylation levels (data not shown). Overall, the following 4 cell types were established and used in the in vitro experiments: p53^shRNA^, Ras^G12V^, Ras^G12V^ + p53^shRNA^ and control cells (expressing control vectors for both types of transfection). For use in other in vitro experiments, cells transiently expressing GFP-H-WT-Ras (=WT-Ras) have been generated (all procedures were performed as detailed above for GFP-H-Ras^G12V^). For in vivo experiments, MCF-7 cells were infected to express H-Ras^G12V^ or control vector (p-Babe). Then, stable cells were selected by 50 μg/ml hygromycin and Ras^G12V^ over-expression was verified by quantitative real-time polymerase chain reaction (qRT-PCR; Data not shown).

Also, transient transfections with ErbB2 were performed (vector kindly provided by Prof. Pinkas-Kramarski, Tel Aviv University, Tel Aviv, Israel). ErbB2 over-expression was verified by qRT-PCR (see “Results”), and the whole population of transiently-transfected cells was used.

In specific experiments, a pool of 4 siRNAs to p65 (Cat # MU-003533-02; Dharmacon, Lafayette, CO, USA) or control siRNA (Dharmacon) were introduced to the cells by ICAFectin (Cat. # ICA441; In-Cell-Art, Nantes, France, following manufacturer’s instructions), together with WT-Ras. After this step (that by definition cannot be followed by selection), the cell population was used as a whole, and effective p65 down-regulation was verified by WB (see “Results”).

### ELISA assays and qRT-PCR analyses

**Figure 1:**
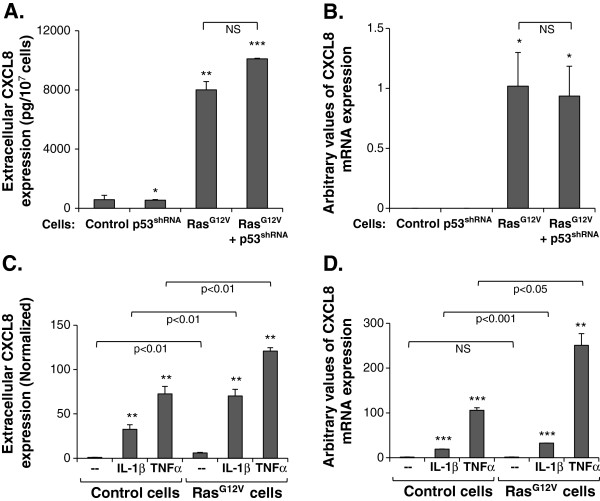
**Ras**^**G12V **^**induces CXCL8 expression independently of deregulated p53, and synergizes with the inflammatory cytokines TNFα and IL-1β.** MCF-7 cells were transfected to express p53^shRNA^, Ras^G12V^, Ras^G12V^ + p53^shRNA^ or the appropriate control vectors. **(A, B)** Induction of CXCL8 by Ras^G12V^ ± p53^shRNA^ expression, determined in cell CM at the protein level by ELISA **(A)**, or at the mRNA level by qRT-PCR **(B)**. **(C, D)** Induction of CXCL8 expression by the synergistic activities of Ras^G12V^ with IL-1β (500 pg/ml) or TNFα (50 ng/ml), determined at the protein level by ELISA **(C)**, and at the mRNA level by qRT-PCR **(D)**. Cytokine concentrations were selected based on previous titration analyses. *p < 0.05, **p < 0.01, ***p < 0.001 compared to control transfectants **(A, B)**, or to non-stimulated cells **(C, D)**. NS = Not significant. In all panels, a representative experiment of n≥3 is presented. Please see “Methods” for additional details on times of CM collection, and of mRNA analyses.

**Figure 2:**
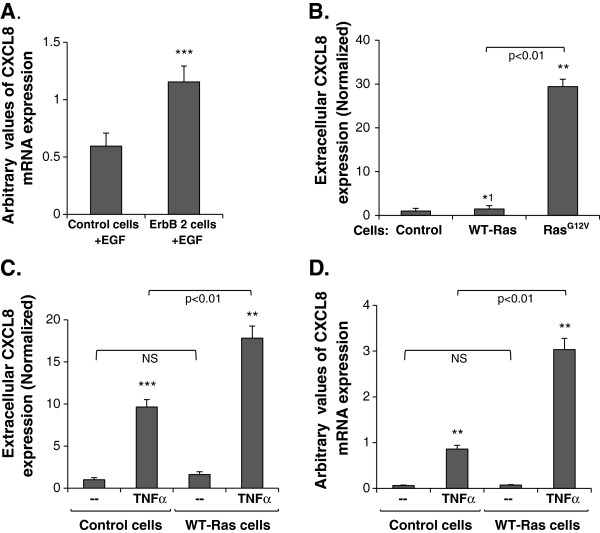
**TNFα and WT-Ras cooperate in inducing CXCL8 up-regulation. (A)** Induction of CXCL8 at the mRNA level, determined by qRT-PCR in MCF-7 cells transfected to over-express ErbB2 or control vector, and stimulated by EGF (30 ng/ml). **(B)** Induction of CXCL8 at the protein level, determined by ELISA in CM of MCF-7 cells transfected to express Ras^G12V^, WT-Ras or the appropriate control vector. **(C, D)** CXCL8 induction in MCF-7 cells transfected to express WT-Ras and stimulated by TNFα (50 ng/ml), determined at the protein level in cell CM by ELISA **(C)** and at the mRNA level by qRT-PCR **(D)**. *p < 0.05, **p < 0.01, ***p < 0.001 compared to control transfectants **(A, B)**, or to non-stimulated cells **(C, D)**. ^1^, Not in all assays this value was significant. NS = Not significant. In all panels, a representative experiment of n≥3 is presented. Please see “Methods” for additional details on times of CM collection, and of mRNA analyses.

**Figure 3:**
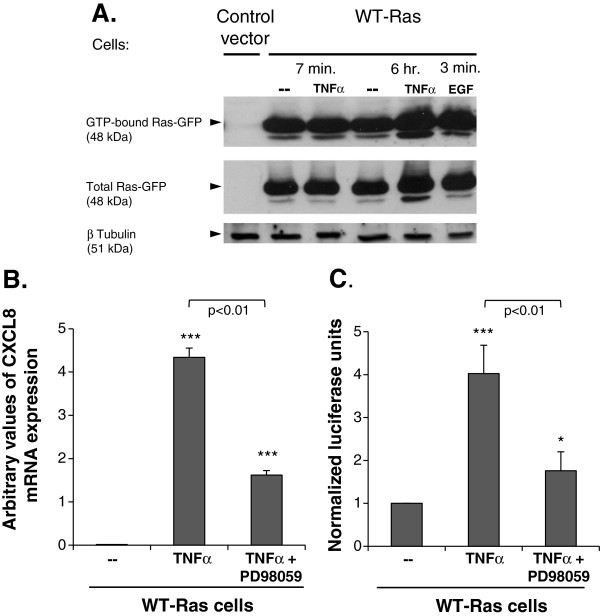
**TNFα stimulation leads to increased expression of active GTP-bound WT-Ras, together giving rise to CXCL8 up-regulation through the MEK pathway. (A)** MCF-7 cells were transfected to express Ras^G12V^, WT-Ras or the appropriate control vector. Cell lysates were used for RBD pull-down assays, determining the levels of activated GTP-bound Ras, and in parallel for determination of total Ras or β tubulin (loading control). The figure shows the levels of GTP-bound Ras in WT-Ras-transfected cells, not-stimulated or stimulated by TNFα (50 ng/ml; 7 min or 6 hr) or EGF (100 ng/ml; 3-4 min). The figure also shows that Ras was not detected in cells transfected with the empty control vector. The fast-migrating band of GTP-bound Ras has been detected by others [49-53], and may represent a post-translationally modified form of the protein. This band was highly expressed in the Ras^G12V^-expressing tumor cells, and also could be minimally detected in WT-Ras-expressing tumor cells, albeit only following longer exposure (Additional file 3A). **(B, C)** MCF-7 cells that were transfected to express WT-Ras were not-stimulated or stimulated by TNFα (50 ng/ml) in the absence or in the presence of the MEK inhibitor PD98059 (50 μM). **(B)** CXCL8 mRNA levels were determined by qRT-PCR. **(C)** CXCL8 expression levels were determined by dual luciferase assay, using the luciferase gene under the control of WT CXCL8 promoter. Non-stimulated cells were given the value of 1. In panels **A-B** a representative experiment of n≥3 is presented. Panel **C** presents the average ± SD of n=3. *p<0.05, ***p<0.001 compared to non-stimulated cells. In Panel **A**, the EGF results are representatives of 3 out of 4 stimulations performed. Please see "Methods" for additional details on the experimental procedures and statistical analyses performed in this part of the study.

**Figure 4:**
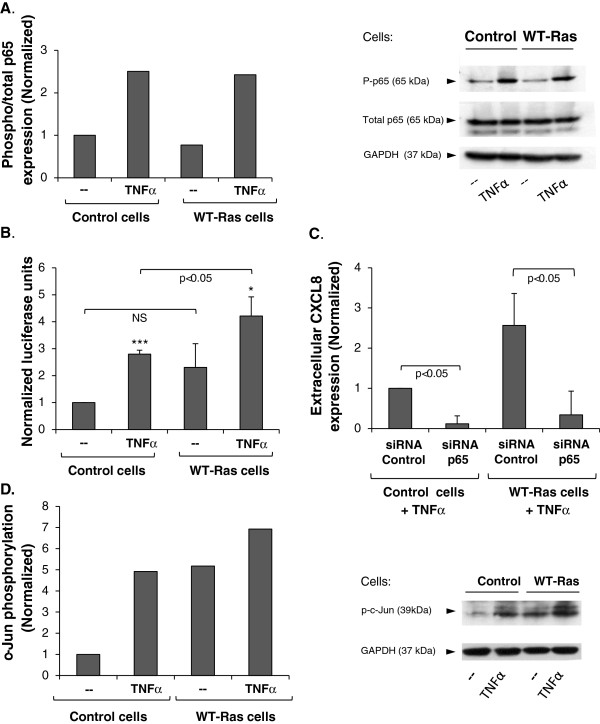
**TNFα + WT-Ras up-regulate CXCL8 expression via the activation of NF-κB and induce AP-1 stimulation.** MCF-7 cells were transfected with WT-Ras vector or with control vector, and were not-stimulated or stimulated by TNFα (50 ng/ml). **(A)** p65 phosphorylation was determined by WB. Control vector-transfected non-stimulated cells were given the value of 1. **(B)** NF-κB activation was determined in cells transfected to express the luciferase gene under the control of 3 conserved repeats of NF-κB binding sites, using dual luciferase assay. Control vector-transfected non-stimulated cells were given the value of 1. The results obtained in each of the 3 repeats are presented in Table 1. **(C)** WT-Ras-expressing cells were transfected with a pool of 4 siRNAs targeting p65 (25-35 nM), or with appropriate control siRNA. CXCL8 protein expression levels were determined in cell CM by ELISA. **(D)** c-Jun phosphorylation was determined by WB, following c-Jun immunoprecipitation. GAPDH was used for determination of protein amounts in original cell lysates, prior to immunoprecipitation. Control vector-transfected non-stimulated cells were given the value of 1. The direct roles of AP-1 in mediating the TNFα + WT-Ras stimulation of CXCL8 are presented in Table 2. In panels **A** and **D** a representative experiment of n≥3 is presented. Each of the results presented in Panels **B** and **C** show the average ± SD of n=3. *p < 0.05, ***p < 0.001 compared to non-stimulated cells. Please see “Methods” for additional details on the experimental procedures and statistical analyses performed in this part of the study.

Following transfection with vectors coding for Ras^G12V^, WT-Ras, p53^shRNA^ or with control vectors, MCF-7 cells were grown in serum-free medium. Based on titration analyses, the cells were stimulated with TNFα or IL-1β at selected concentrations, which agree with the conventional concentration range used in other research systems: recombinant human (rh) TNFα at 50 ng/ml (Cat. # 300-01A; PeproTech, Rocky Hill, NJ, USA), rhIL-1β at 500 pg/ml (Cat. # 200-01B; PeproTech), or their solubilizer (0.1% BSA). Chemokine secretion and mRNA levels were determined by ELISA and qPCR analyses (Figures 1,2,3,4).

For ELISA assays, the cells were grown in serum-free medium for 24 hr without or with cytokine stimulation. Then, CXCL8 and CCL2 levels were determined by ELISA in conditioned medium (CM), using standard curves with rhCXCL8 or rhCCL2 (Cat. # 200-08 or # 300-04, respectively; PeproTech), at the linear range of absorbance. The following antibodies were used (all from PeproTech): For CXCL8 - coating monoclonal antibodies (Cat. # 500-P28), detecting biotinylated rabbit polyclonal antibodies (Cat. # 500-P28Bt); For CCL2 - coating monoclonal antibodies (Cat. # 500-M71), detecting biotinylated rabbit polyclonal antibodies (Cat. # 500-P34Bt). Then, streptavidin-horseradish peroxidase (HRP; Jackson ImmunoResearch Laboratories, West Grove, PA) and the substrate TMB/E solution (Chemicon, Temecula, CA, USA) were added. The reaction was stopped by the addition of 0.18 M H_2_SO_4_ and was measured at 450 nm.

In general, chemokine mRNA levels were determined by qRT-PCR at the termination of the experiment, when CM were collected for ELISA. In specific cases (Figures 1D and 2A), mRNA levels were determined after 6-8 hr following cell stimulation, based on kinetics analyses. Total RNA was isolated from the cells using the EZ-RNA kit (Biological Industries), and first-strand cDNA was produced using the M-MLV reverse transcriptase (Ambion, NY, USA). Quantification of cDNA targets by qRT-PCR was performed on Rotor Gene 6000 (Corbett Life Science, Sydney, Australia), using Rotor Gene 6000 series software. Transcripts were detected using SYBR Green I (Thermo Fisher Scientific, Waltham, MA, USA) according to the manufacturer’s instructions. The primers were as follows: For CXCL8 (Genbank accession no. NM_000584): forward 5′-TTCTGCAGCTCTGTGTGAAG-3′, reverse 5′-CAGTGTGGTCCACTCTCAAT-3′; For CCL2 (Genbank accession no. NM_002982): forward 5′-TCGCTCAGCCAGATGCAATC-3′, reverse 5′-CCTTGGCCACAATGGTCTTG-3′; For ErbB2 (Genbank accession no. NM_001005862): forward 5′-GAAACCTGACCTCTCCTACATG-3′, reverse 5′-TTGTCATCCAGGTCCACACA-3′; For the normalizing gene rS9 (Genbank accession no. NM_001013): forward 5′-TTACATCCTGGGCCTGAAGAT-3′ and reverse 5′-GGGATGTTCACCACCTGCTT-3′. PCR amplification was performed over 40 cycles (95°C for 15 seconds, 59°C for 20 seconds, 72°C for 15 seconds). Dissociation curves for each primer set indicated a single product, and no-template controls were negative after 40 cycles. Quantification was performed by standard curves, on the linear range of quantification.

When indicated, the pharmacological inhibitor of MEK, PD98059 (Cat. # 9900; Cell signaling Technology, Danvers, MA, USA), was used in a conventional concentration of 50 μM. The inhibitor was added to cell cultures 2 hr prior stimulation of the cells by TNFα, and was present in culture throughout the duration of stimulation. Control cells were treated with the solubilizer of the drug at similar dilution (Dimethyl sulfoxide, DMSO; Sigma, Saint Louis, MO).

### Determination of GTP-Ras levels by Ras-binding-domain assays

Cells grown in serum-free medium were stimulated by TNFα (50 ng/ml) or epidermal growth factor (EGF; 100 ng/ml) for time points indicated in the relevant figures. Cell lysates were used in two parallel procedures (Figure 3): (1) GTP-Ras levels were determined by the glutathione S-transferase-Ras-binding-domain of Raf (RBD) pull-down assay as previously described [46], followed by determination of activated Ras levels by pan-anti-Ras antibodies (Cat. # OP40; Calbiochem, Gibbstown, NJ, USA) using WB. (2) Equivalent total lysates were used to determine total Ras levels (antibody as above) and β-tubulin (Cat. # AK-15; Sigma) by WB.

### WB analyses

Cells grown in serum-free medium were stimulated by TNFα (50 ng/ml) for 5 and 10 min in studies of Erk phosphorylation, for 10 min in NF-κB stimulation or for 30 min in c-Jun activation (based on kinetics analyses). To detect decrease in IκBα - the NF-κB inhibitor whose degradation allows for p65 activation - the levels of IκBα were determined following 24 hr of stimulation by TNFα (based on previous kinetics analyses).

Following stimulation, cells were lysed in RIPA lysis buffer. Lysis was followed by conventional WB procedures. Antibodies against the following proteins were used: phosphorylated Erk (Cat. # M9692; Sigma); Erk (Cat. # M5670; Sigma), p53 (From DO-1 hybridoma, kindly provided by Prof. Sara Lavi, Tel Aviv University, Tel Aviv, Israel); phosphorylated p65 (Cat. # 3033; Cell Signaling Technology); total p65 (Cat. # 4764; Cell Signaling Technology); IκBα (Cat. # 4814; Cell Signaling Technology); GAPDH (Cat. # ab9485; Abcam, Cambridge, UK). Phosphorylated c-Jun was immunoprecipitated and detected by antibodies targeting phosphorylated c-Jun (Cat. # 1527-S; Epitomices, Burlingame, CA, USA); Ras and tubulin antibodies – please see below in the following sub-section.

After transfer to membranes, HRP-conjugated secondary antibodies were used, as appropriate: goat anti-mouse-HRP (Cat. # 115-035-166; Jackson ImmunoResearch Laboratories, West Grove, PA, USA) and goat anti-rabbit-HRP (Cat. # 111-035-003; Jackson ImmunoResearch Laboratories). The membranes were subjected to enhanced chemiluminescence, and bands on immunoblots were quantitated by densitometry using TINA image analysis software.

### Dual luciferase assays

The assays were performed with firefly luciferase gene under the control of the following promoters: (1) WT CXCL8 promoter (Figure 3). (2) Promoter expressing 3 conserved NF-κB binding sites (3X-κB-L, including MHC NF-κB binding sites), kindly provided by Prof. Wiemann (DKFZ, Heidelberg, Germany) (Figure 4 and Table 1). (3) CXCL8 promoter expressing WT or mutated AP-1 binding site (Table 2). The promoter included the 5′-flanking region from -558 to +98 bp, with WT AP-1 binding site (5′-AAGTGTGATGACTCAGGTTTGCCCTGA-3′) or AP-1-mutated binding site (5′-AAGTGTGATATCTCAGGTTTGCCCTGA-3′). Both constructs were kindly provided by Prof. Muhl (University Hospital Goethe-University, Frankfurt, Germany). In each case, a construct coding for renilla luciferase was used for normalization of the results according to transfection yields (kindly provided by Dr. Zor, Tel Aviv University, Tel Aviv, Israel).

**Table 1:** **The cooperativity of WT-Ras and TNF**α **stimulates the transcriptional activity of NF-κB**

	**Control cells**		**WT-Ras cells**	
	**–**	**TNFα**	**–**	**TNFα**
**Exp #1**	1.00	2.64	1.62	3.39
**Exp #2**	1.00	2.94	3.29	4.64
**Exp #3**	1.00	2.78	1.98	4.60

MCF-7 cells were transfected with WT-Ras vector or with control vector, and were not-stimulated or stimulated by TNFα (50 ng/ml). Stimulation of the transcriptional activity of NF-κB was determined in cells transfected to express the luciferase gene under the control of 3 conserved repeats of NF-κB binding sites, using dual luciferase assay. Control vector-transfected non-stimulated cells were given the value of 1. The table presents the results obtained in 3 independent experiments, whose average results are shown in Figure 4B. Please see “Methods” for additional details on the experimental procedures performed in this part of the study.

**Table 2:** **TNF**α **+ WT-Ras up-regulate CXCL8 expression via the activation of AP-1**

	**Control cells**				**WT-Ras cells**			
	**WT CXCL8 promoter**		**AP-1 mutated CXCL8 promoter**		**WT CXCL8 promoter**		**AP-1 mutated CXCL8 promoter**	
	–	TNFα	–	TNFα	–	TNFα	–	TNFα
**Exp #1**	1.00	1.56	0.03	0.12	0.72	2.42	0.05	0.14
**Exp #2**	1.00	5.40	0.10	0.42	1.79	6.11	0.12	0.48
**Exp #3**	1.00	3.33	0.11	0.30	1.05	4.94	0.07	0.16

MCF-7 cells were transfected with WT-Ras vector or with control vector, and were not-stimulated or stimulated by TNFα (50 ng/ml). Stimulation of the transcriptional activity of AP-1 was determined in cells transfected to express the luciferase gene under the control of WT AP-1, or mutated AP-1 binding sites in the CXCL8 promoter, using dual luciferase assay. Control vector-transfected non-stimulated cells were given the value of 1. The table presents the results obtained in 3 independent experiments. Please see “Methods” for additional details on the experimental procedures performed in this part of the study.

In luciferase assays, all relevant vectors (including WT-Ras) were transiently transfected to MCF-7 cells by ICA Fectin. After 24 hr, the cells were stimulated by TNFα for 8 hours in serum-free medium (on the basis of preliminary kinetics studies) to allow for promoter activation, and were processed with the reagents provided in the Dual-Luciferase Assay System Kit (Cat. # E1019; Promega, Madison, WI, USA). Luciferase activity was determined using the same kit according to the manufacturer’s instructions. When indicated, the MEK inhibitor PD98059 was used, under the same conditions described above.

### Chick chorioallantoic membrane (CAM) assay

**Figure 5:**
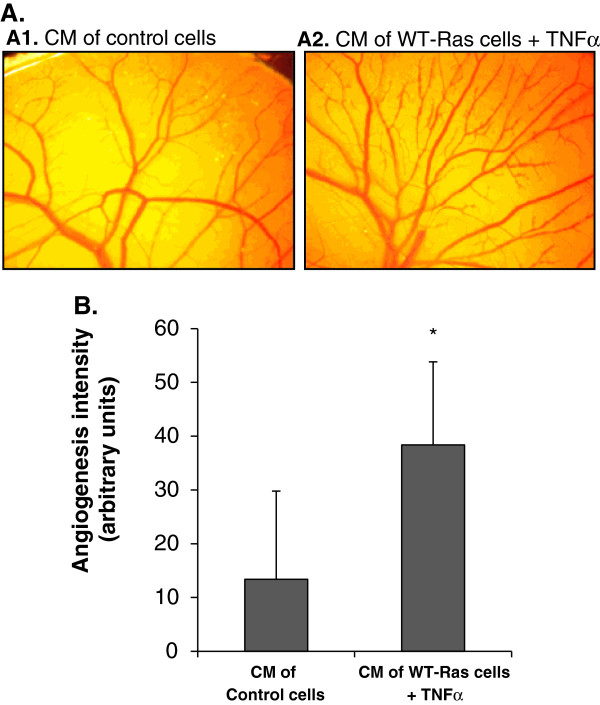
**CM of TNFα-stimulated WT-Ras-expressing cells lead to increased angiogenesis.** CM of MCF-7 cells were administered on chick chorioallantoic membranes (CAM), in which length, thickness and sprouting of embryo vessels were used to determine angiogenicity. Two types of CM were used (see "Results" for details): (1) From non-stimulated control cells; (2) From WT-Ras-expressing cells, stimulated by TNFα (50 ng/ml). **(A)** A representative CAM image. In each group, n≥5 embryos were tested, in each of 3 independent experiments. **(B)** In two of the experiments, angiogenesis intensity was determined by three researchers in an unbiased manner, using parameters of length, thickness and sprouting of embryo vessels, combined. In each of the two independent experiments, n≥5 embryos were tested in each group. Please see "Methods" for additional details on times of CM collection.

For assessment of neo-vascularization, WT-Ras over‒expressing cells were stimulated by TNFα (50 ng/ml) in serum‒free medium, while vector-expressing control cells were not treated with TNFα. After 24 hr (allowing for accumulation of angiogenic factors), CM were collected and used in CAM assays (Figure 5). To this end, 25 mm^2^ gelatin patches were soaked in the CM for 4 hr, and then implanted on the top of the growing CAM on embryonic day 3 of development. Patches were replaced on a daily basis for the following 3 days of the experiment. On embryonic day 6, angiogenesis intensity was determined on the basis of length, thickness and sprouting of the embryo vessels, combined. Angiogenesis was evaluated independently by 3 researchers in an unbiased manner. Pictures were taken using a camera set on a binocular.

### Flow cytometry

Transfection yields of GFP-Ras^G12V^ and GFP-WT-Ras were determined by flow cytometry, using a Becton Dickinson FACSort (Mountain View, CA, USA). Baseline staining was obtained by using untransfected cells. Staining patterns were determined using the win MDI software.

### Tumor growth and metastasis

In these assays we used MCF-7 cells that were infected to stably express Ras^G12V^, or cells infected by control vector (previously described in “Cells, vectors and transfections”). Then, these cells were infected to stably express mCherry (by pQC-mCherry retroviral vector). mCherry + Ras^G12V^ -expressing cells, or mCherry-control cells, were either not-stimulated or stimulated by TNFα (50 ng/ml) for 8 hr, then the medium was exchanged to a serum-deprived medium, without TNFα. After additional 16 hr that allowed TNFα-induced intracellular processes to take place, the cells were inoculated to the mammary fat pad of female nude mice, as described in Figure 3A.

**Figure 6:**
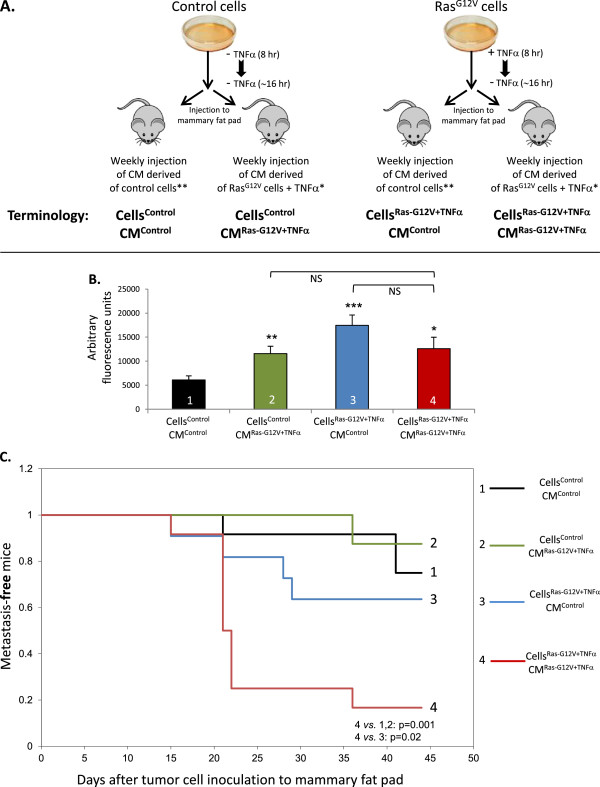
**Cooperativity between TNFα and hyper-activated Ras promotes the dissemination of tumor cells to lymph nodes.** The scheme describes the "Experimental design" of in vivo mouse experiments, including cell preparation. *CM preparation: Ras^G12V^**(A)** MCF-7 cells were stimulated by TNFα for 8 hr, CM were removed and replaced by fresh, serum-free non-TNFα-containing medium for additional 36 hr. **CM preparation: The same as in *, but no TNFα included at any stage. **(B)** Determination of tumor growth in the mammary fat pad of mice. All MCF-7 tumor cells expressed mCherry, to enable their detection by the Maestro device in intact mice. To provide accurate determination of tumor sizes, the Maestro device was used to quantify fluorescence in excised tumors, ex vivo, at the end of two experiments performed (termination of experiments was based on animal care regulations). The Figure shows combined results of these experiments, including n=7 in each of the mice groups. For more details on the results in group 4 – see "Results". *p<0.05, **p=0.01, ***p=0.001 for comparisons between the Cells^Control^CM^Control^ group and all other groups. **(C)** Kinetics of tumor cell dissemination to LN, followed by the Maestro device in intact mice in three independent experiments combined, including a total of n=10-12 in each of the mice groups. All tumor cells expressed mCherry, to enable their detection by the Maestro device in intact mice, p values are shown in the Figure. No statistical differences were obtained in comparisons between any of the other Cell-CM combinations.

Ten days prior to tumor cell injection to female nude mice, the mice were implanted sub-cutaneously with slow-release estrogen pellets (1.7 mg/pellet, 60 days slow release, SE-121; Innovative Research of America, Sarasota, FL, USA). The different mCherry-expressing tumor cells (4×10^6^/mouse) were supplemented with matrigel (Cat. # 356234; BD Biosciences, Franklin Lakes, NJ, USA) and CM that were mixed in 1:1 volume (see Figure 6A for details). The cells were injected to the mammary fat pad of mice, and once a week the mice were injected intra-tumor with 150 μl CM (concentrated ~×12), obtained from control cells or TNFα-stimulated Ras^G12V^ -expressing cells, as described in Figure 6A.

Tumor progression and LN metastases were monitored weekly by CRI™ Maestro non-invasive intravital imaging system in intact mice. At the termination of the experiments (see legend to Figure 6B), tumors were excised and their size was analyzed by the Maestro device. Due to depth of the lung tissue, mCherry signals in the lungs were not well detected by the Maestro device when intact mice were analyzed. Therefore, kinetics of lung metastases were not followed in the study. The regulations of Tel Aviv University Animal Care Committee did not allow continuation of the experiments to the stage of survival analysis. All procedures involving experimental animals were performed in compliance with local animal welfare laws, guidelines and policies.

### Statistical analyses

Statistical analyses of in vitro experiments were done using Student’s t tests. Values of p < 0.05 were considered statistically significant, and data were presented as mean ± SD. In the in vivo studies of primary tumors, statistical analyses of tumor size were done using Student’s t tests, and values of p < 0.05 were considered statistically significant. The data were presented as mean ± SEM. Analyses of kinetics of metastasis-free mice were done using Kaplan-Meier’s method, and comparison between groups was tested by log-rank test. Values of p < 0.05 were considered statistically significant. Adjustment for multiplicity of comparisons was done using the Benjamini-Hochberg procedure. Using this procedure, all the significant results that were presented in the manuscript remained statistically significant after correcting for their multiplicity [except for Figure 1B and Figure 6B (comparison between groups 4 and 1). Also, several of the results in Figure 4B did not remain statistically significant after the correction because the intensity of response varied between the different experiments. Therefore, to show the reproducibility of the results the data were also provided in Table 1].

### Data presentation

All the in vitro experiments were repeated at least 3 times with similar results. The results of most studies were presented as a representative experiment of such similar repeats. Alternatively, when more appropriate to the experimental conditions of the assays (e.g., luciferase tests), the results were presented as average of at least n=3.

## Results

### In breast tumor cells, Ras^G12V^ induces CXCL8 (and CCL2) without need for cooperative down-regulation of p53

At the beginning of this study, we asked whether tumor cells express similar regulatory patterns to those of non-transformed cells [9], in terms of CXCL8 regulation by tumor-promoting alterations in Ras and p53. To address this question we performed the analyses with MCF-7 cells. These cells are human luminal breast tumor cells like the majority of tumors in breast cancer patients, they express WT-p53 [30,42], and do not carry mutations in Ras as is the case in most human breast tumors [1,29,30,47]. These cells also respond to TNFα and IL-1β, which were introduced in the proceeding stages of the study. Thus, MCF-7 cells provided an ideal platform to conduct our studies (that could not be recapitulated in other luminal human breast tumor cells because they did not carry identical properties to those of MCF-7 cells in terms of p53 expression, Ras and ErbB2 activation or the expression of relevant signaling pathways [30,42]).

To address the roles of p53 in CXCL8 regulation, stable transfectants were produced, in which the tumor-suppressor p53 was down-regulated by shRNA (p53^shRNA^; Additional file 1A). In parallel, the cells have undergone transient over-expression with the constitutively active GFP-tagged Ras^G12V^ protein (High Ras^G12V^ expression levels were verified as shown in Additional file 1B; Ras^G12V^ activation has been validated by RBD assays that are described below and by Erk phosphorylation tests whose data are not shown). By taking this general approach of Ras hyper-activation, we have recapitulated the excessive activation of the Ras pathway in breast cancer, which is induced in patients by multiple RTK ligands such as epidermal growth factor (EGF) [1,27,28,47,48]. Overall, the following 4 cell types were established and used in the in vitro experiments: p53^shRNA^, Ras^G12V^, Ras^G12V^ + p53^shRNA^ and control cells (expressing the two control vectors). Of note, to follow on the results described with Ras^G12V^, in more progressed stages of the study, WT-Ras was also addressed (see below).

Similarly to the findings obtained in non-transformed cells [9], Ras^G12V^ + p53^shRNA^ had induced the expression of CXCL8 in breast tumor cells (Figures 1A and B). However, in contrast to the non-transformed cells [9], Ras^G12V^ was fully active on its own in inducing CXCL8 in the tumor cells, at the protein and mRNA levels (Figure 1A and B, respectively), while p53^shRNA^ alone did not induce any change in chemokine expression, and did not add significantly to CXCL8 up-regulation by Ras^G12V^ (Figure 1A and B).

These data indicate that in the tumor cells, constitutively active Ras^G12V^ could act alone to up-regulate the expression of CXCL8, with no need for cooperativity with p53 deregulation. Similar findings were obtained for CCL2 (Additional file 2), another member of the “cancer-related chemokine cluster” that was addressed in our previous study of non-transformed cells [9]. These observations contrasted the findings in non-transformed cells, where Ras^G12V^ had to cooperate with down-regulation of p53 in order to induce CXCL8 and CCL2 up-regulation [9]. This difference between the non-transformed and malignant cells may be due to discrepancies in their genetic setup, as will be discussed further below (“Discussion”).

### In breast tumor cells, inflammatory cytokines act in a cooperative manner with Ras^G12V^, together giving rise to exacerbated expression of the pro-angiogenic chemokine CXCL8

The above findings were followed by determination of the impacts imposed by inflammatory mediators on the expression of CXCL8. To this end, the tumor cells were stimulated by TNFα or IL-1β, using selected concentrations based on previous titration analyses. The results of Figure 1C indicate that stimulation by TNFα or IL-1β has induced a prominent up-regulation of CXCL8 secretion, and moreover, that both cytokines acted in a synergistic manner with Ras^G12V^, leading to exacerbated release of CXCL8 by the cells. The basis for the cooperative activities of Ras^G12V^ with the two cytokines was in increased mRNA levels (Figure 1D; Please note that up-regulation in CXCL8 mRNA expression in control non-stimulated Ras^G12V^-expressing cells could not be detected technically under these experimental conditions because of the very high induction of CXCL8 mRNA in Ras^G12V^-expressing cells that were stimulated with TNFα and IL-1β).

Thus, hyper-activated Ras^G12V^ cooperated with inflammatory factors that were shown to be prevalent at the breast tumor microenvironment [21], together potentiating the release of the powerful angiogenic and tumor-promoting chemokine CXCL8 by the tumor cells. However, in breast tumors, Ras is rarely mutated, but nonetheless it is continuously activated because of excessive stimulation of RTKs such as ErbB2 [1,27,28,47,48]. This would mean that in breast tumor cells that express endogenously WT-Ras, CXCL8 may be induced by RTK ligands. To see if this is indeed the case, we have used the ErbB2-EGF axis as a proof of concept, with ErbB2-over-expressing MCF-7 cells (Additional file 3A; At the basal level, MCF-7 cells express relatively low levels of the receptor [45]). In these cells, EGF stimulation has induced the expression of CXCL8 (Figure 2A), indicating that activation of RTKs is a relevant pathway for induction of CXCL8, which may account for Ras hyper-activation in breast tumor cells that do not carry mutated Ras.

### TNFα cooperates with WT-Ras in elevating CXCL8 levels, and promotes the expression of activated GTP-bound WT-Ras

Noting that WT-Ras is the form of the protein that is abundant in most breast tumor cells [1,47], we asked whether it acts similarly to Ras^G12V^, and if it is able to act alone to induce CXCL8 up-regulation. To study the regulatory functions of a protein that is endogenously expressed in a WT form in the cells, one needs to either decrease or increase the expression levels of the protein, and determine the effects of such manipulations on the issue that is addressed. Because MCF-7 cells express three different WT isoforms of Ras [29], the down-regulation approach would require efficient reduction in the expression of all three Ras variants without perturbing cellular growth, and such a process may be difficult to achieve. Therefore, we chose an alternative attitude in which we over-expressed WT-Ras in the cells. This latter approach, which is conventionally used as the method-of-choice in many studies of Ras, also enabled us to adequately compare WT-Ras to Ras^G12V^, which has been studied in the previous parts of this work.

Thus, WT-Ras was over-expressed in the cells (e.g., Additional files 3B and C), and CXCL8 expression levels were determined. Unlike Ras^G12V^, the over-expression of WT-Ras in the tumor cells did not induce the expression of CXCL8 (Figure 2B). However, when WT-Ras-expressing tumor cells were stimulated by TNFα, cooperativity between the two pathways was obtained. This was indicated by the fact that CXCL8 was not induced by WT-Ras expression alone but was highly promoted when WT-Ras expressing cells were stimulated by TNFα. This elevated response was evidenced at the protein and mRNA levels (Figures 2C and D, respectively).

These results attest for functional cooperativity between TNFα and WT-Ras, leading to induction of CXCL8 expression as was the case when Ras^G12V^ was expressed in the cells. These findings suggest that stimulation by TNFα has led to activation of WT-Ras, which was not active otherwise. In such a case, TNFα stimulation was expected to lead to increased levels of activated WT-Ras, at the molecular level. To test this possibility, we established the methods for detecting Ras activation, using Ras^G12V^ - which is the constitutively active form of the protein - as a positive control. To determine the levels of Ras activation, we used RBD pull-down assays that give rise to GTP-bound Ras, which is well-established as the activated form of the protein [1,2,49]. As shown in Additional file 3A, large amounts of GTP-bound Ras indeed have been observed in cells expressing our positive control of Ras^G12V^, while no detection of Ras was obtained in control vector-expressing cells, as expected (Figure 3A). The GFP-tagged GTP-bound Ras was observed in the expected MW of ~48 kDa, and the fast migrating band of GTP-bound Ras^G12V^ detected in this case may represent a post-translational modification of Ras which was observed by others in analyses of H-Ras and of other forms of Ras [49-53] (please note that in this experiment, the fast migrating band was detected, albeit in very low levels, also in non-stimulated WT-Ras-expressing cells. Its detection required longer film exposure, as shown in Additional file 3C).

When the levels of activated Ras were compared between Ras^G12V^ and WT-Ras, we found that following the RBD pull-down assays the levels of GTP-bound WT-Ras were smaller than those of GTP-bound of Ras^G12V^. These differences between Ras^G12V^ and WT-Ras agree with the fact that Ras^G12V^ is the constitutively active form of the protein and with our previous observations (Figure 2B), showing that Ras^G12V^ induced CXCL8 up-regulation, while WT-Ras did not (in the absence of TNFα stimulation).

Then, we determined the impact of TNFα on the expression levels of activated GTP-bound WT-Ras. We found that stimulation of WT-Ras-expressing cells with TNFα for 6 hr has led to up-regulation in the amounts of activated WT-Ras obtained by the RBD pull-down assays (Figure 3A), as was the case also following the activation of WT-Ras-expressing cells by an EGF control (stimulatory conditions adhering to previously published studies of Ras activation by EGF [54-56]; Figure 3A). Thus, TNFα has induced the activation of WT-Ras, in a process that was time-dependent (it was not induced by brief stimulation with TNFα for 7 minutes), suggesting that the cytokine has induced autocrine mechanisms leading to up-regulation of activated WT-Ras. Here, we would like to indicate that endogenous WT-Ras probably did not account much to the response induced in the cells by TNFα stimulation. MCF-7 cells express relatively small quantities of endogenous WT-Ras, particularly following RBD pull-down assays in experiments detecting GTP-bound Ras (Additional file 3D; Endogenous WT-Ras had the expected MW of 21 kDa), and the protein levels were different within experiments. However, we found that WT-Ras over-expression provided a biologically relevant system because in some of the experiments we could detect a certain increase in the levels of activated GTP-bound endogenous WT-Ras after TNFα activation (but their levels were low relatively to the amounts obtained by the over-transfected WT-Ras; Because of the low detectability of endogenous Ras, the relevant data were not shown).

The above findings obtained with TNFα-activated, over-expressed, WT-Ras indicate that in response to TNFα, WT-Ras has been activated at the molecular level and has gained functional properties similar to those of Ras^G12V^. This was manifested also by the ability of TNFα-activated WT-Ras to induce increased expression of CXCL8, as did Ras^G12V^. Supporting a mechanism in which WT-Ras has been turned into an active entity, and in line with the fact that the MEK-Erk pathway mediates many of the Ras-induced activities [1], MEK-dependent pathways were involved in the ability of TNFα to induce CXCL8 expression in WT-Ras-expressing tumor cells. The inhibition of the down-stream effects of MEK by the MEK-inhibitor PD98059 (evidenced by inhibition of Erk1 and Erk2 activation in Additional file 4), has led to prominent reduction of CXCL8 expression (at the mRNA level; Figure 3B), and to potent inhibition in luciferase expression in CXCL8 promoter-luciferase reporter assays (Figure 5C).

Thus, our findings indicate that following TNFα stimulation, the content of active, GTP-bound WT-Ras was increased, recapitulating the activation state of Ras^G12V^ and leading to increase in the release of CXCL8, a highly angiogenic and pro-malignancy factor. These results indicate that TNFα has turned WT-Ras into an activated, tumor-promoting entity.

### The synergistic activities of WT-Ras and TNFα on CXCL8 up-regulation are mediated by the NF-κB and AP-1 transcription factors

Throughout this study, we found that CXCL8 up-regulation took place at the mRNA level (Figures 1B,D and 2D). Therefore, we asked which regulatory elements are inducing the transcription of CXCL8, thus leading to the ability of TNFα + WT-Ras to eventually promote CXCL8 secretion. Here, we studied the roles of NF-κB and AP-1, two transcription factors known to up-regulate CXCL8 in the immune context, although to different extents depending on cell type and stimulus [57].

The activation of NF-κB comes into effect following down-regulation of the IκBα inhibitor and phosphorylation of p65 (RelA) [58]. Following TNFα stimulation, the phosphorylation of p65 was increased (Figure 4A) and IκBα levels were reduced (Additional file 5A). These general assays of NF-κB activation did not reveal cooperativity between TNFα and WT-Ras. However, more direct and sensitive analyses with dual luciferase assays using the NF-κB-luciferase reporter, demonstrated that the stimulation of WT-Ras-expressing cells by TNFα has increased the transcriptional activity of NF-κB (Figure 4B and Table 1). Also, siRNAs to p65 have down-regulated p65 expression (Additional file 5B), and in cells stimulated by TNFα have led to almost complete shut-off of the TNFα + WT-Ras-induced CXCL8 expression (at the protein level; Figure 4C). These results provide evidence for direct roles of the NF-κB pathway in mediating the TNFα + WT-Ras-induced activation of CXCL8.

In parallel, we found that TNFα + WT-Ras induced cooperative induction of c-Jun phosphorylation (Figure 4D), which is a major component of the AP-1 transcription factor. The phosphorylation of c-Jun indicates that there was a general process of AP-1 activation but it could not tell us whether the activation of AP-1 by TNFα + WT-Ras has led directly to up-regulation of CXCL8 expression. Looking for appropriate manners to determine the direct roles of AP-1 in induction of CXCL8 upon TNFα stimulation of WT-Ras-expressing cells, we wished to use siRNA/shRNA to c-Jun; however, we could not obtain efficient enough down-regulation of c-Jun expression, being in line with the fact that c-Jun is essential for cell proliferation [59]. In the absence of a pharmacological inhibitor with high enough specificity, we used luciferase reporter assays in which the CXCL8 promoter expressed WT or mutated AP-1 binding sites. These tests have shown cooperativity between TNFα and WT-Ras in inducing luciferase activation (Table 2); in addition, marked decrease was noted in luciferase levels when WT-Ras cells were stimulated by TNFα in the presence of AP-1-mutated promoter, compared to AP-1-WT promoter (Table 2). Because the promoter was specifically the one of CXCL8, these results demonstrate that TNFα cooperates with WT-Ras in inducing AP-1 activation, together leading to an additive up-regulation in the transcription of CXCL8.

Overall, the results presented in this part of the study indicate that following activation of WT-Ras-expressing cells by TNFα, the NF-κB and AP-1 transcription factors became activated, and led to increased transcription of the CXCL8 gene, and thereafter to increased release of the protein by the tumor cells.

### The functional implications of Ras hyper-activation + TNFα stimulation: Elevated angiogenesis and increased breast tumor cell dissemination to lymph nodes

The results obtained thus far in this study indicate that the cooperative activities of TNFα with Ras^G12V^ or with WT-Ras lead to additive elevation in the release of CXCL8 by the tumor cells. Similarly, many other pro-cancerous factors may be induced in TNFα + Ras-stimulated cells. The outcome of such a process, if taking place in vivo in malignancies with high TNFα expression - as is the case in breast cancer - may be high production of pro-tumorigenic factors by the tumor cells, including angiogenic ones (such as CXCL8 and CCL2).

To examine whether such a general increase in pro-tumoral and angiogenic factors indeed leads to increased angiogenesis, we used the in vivo analysis of chorioallantoic membrane (CAM) assay. In this test, multiple parameters of angiogenesis are affected by angiogenic factors, including length and thickness of blood vessels and their sprouting. Due to its multi-parametric nature, to the high content of vessels in the embryo and to embryo heterogeneity, the results of the CAM assay often show variability between individual samples within the same group; thus, the CAM assay could clearly define differences between two extreme conditions (such as control vs. Ras + TNFα), but its sensitivity could not determine interim effects that may have been obtained by other combinations that are less effective in inducing angiogenic and pro-tumoral factors. To comply with this limitation, and in line with our interest in determining the overall effects induced by multiple angiogenic factors that could have been promoted by the most potent process of TNFα stimulation of WT-Ras-expressing cells, we tested CM from the two most relevant stimulatory extreme conditions: (1) CM of WT-Ras-expressing tumor cells that were stimulated by TNFα. (2) CM of control vector-expressing tumor cells that were not stimulated by the cytokine. The results indicate that CM derived from TNFα-stimulated WT-Ras-expressing tumor cells (shown to produce highly elevated levels of CXCL8; Figure 2C) induced significantly stronger angiogenic effects compared to control cells (Figure 6).

In parallel, we asked what is the impact of combined TNFα stimulation and Ras hyper-activation on tumor growth and metastasis. MCF-7 cells were documented as cells with relatively low malignancy potential, and with very weak invasive and metastasizing capacities [45]. However, published studies by Weinberg and his colleagues have shown that under specific conditions, MCF-7 cells that express oncogenic Ras can form metastases [60]. Thus, to allow for metastatic dissemination in our study, we followed on these observations and used Ras^G12V^-expressing MCF-7 cells, compared to cells transfected with control vector. This approach was valid in our experimental design because of the functional similarities between Ras^G12V^ and TNFα-stimulated-WT-Ras, in terms of Ras activation (Figure 3A and B) and induction of CXCL8 (Figures 1 and 2).

Using these cells as a research platform, we determined the impact of TNFα stimulation and its cooperativity with hyper-activated Ras on the malignancy phenotype of the cells. To this end, two measures were taken (see “Experimental design”, Figure 6A): (1) Ras^G12V^-expressing cells were stimulated by TNFα in vitro before their inoculation to mice in order to induce intracellular mechanisms that would eventually give rise to production of pro-malignancy factors, including CXCL8 (as has been shown in the previous figures of the study). Prior to inoculation to mice, the cells were washed and thus TNFα was removed, in order to prevent a potential acute necrotic effect of TNFα in vivo (such an effect may result out of acute exposure to the cytokine, being in contrast to the chronic and tumor-promoting presence of TNFα at breast tumor sites along disease course). (2) To sustain the in vivo effect of joint TNFα + Ras hyper-activation (Ras^G12V^) in inducing the release of multiple pro-tumorigenic factors by the tumor cells, we have introduced a previously described approach [61,62], in which tumors were inoculated with tumor cell products throughout the process of tumor growth. Here, eight hours following stimulation by TNFα, the medium of the cells was exchanged to TNFα-deficient medium, and following additional 36 hr of cell growth, CM that were enriched in tumor-promoting factors such as CXCL8 (data not shown) were collected and injected to tumors. Thus, tumors were inoculated on a weekly basis with CM derived from TNFα stimulated-Ras^G12V^ cells, compared to CM from control cells. Overall, the analyses included the 4 most relevant groups of mice that could provide insights into the tumor-promoting roles of factors resulting out of the activation of Ras by TNFα (Figure 6A): (1) Cells^Control^CM^Control^; (2) Cells^Control^CM^Ras-G12V+TNFα^; (3) Cells^Ras-G12V+TNFα^CM^Control^; (4) Cells^Ras-G12V+TNFα^CM^Ras-G12V+TNFα^.

Comparison of Cells^Control^CM^Control^ to Cells^Ras-G12V+TNFα^CM^Control^ (groups 1 vs. 3, Figure 6B) has shown that expression of Ras^G12V^ in the cells (stimulated in vitro by TNFα prior to their injection to mice), has led to increased tumor growth. In parallel, CM^Ras-G12V+TNFα^ elevated the ability of Cells^Control^ (cells not expressing Ras^G12V^) to develop primary tumors (groups 2 vs. 1). This latter result indicates that following their stimulation by TNFα, Ras^G12V^-expressing cells secreted to the culture medium soluble factors that had pro-cancerous effects that promoted tumor growth, as was previously indicated by our in vitro analyses of CXCL8 (Figure 1). Cells^Ras-G12V+TNFα^CM^Ras-G12V+TNFα^ also gave rise to bigger tumors than Cells^Control^CM^Control^ (groups 4 vs. 1), but no significant difference was found when the Cells^Ras-G12V+TNFα^CM^Ras-G12V+TNFα^ group was compared to Cells^Ras-G12V+TNFα^CM^Control^ (groups 4 vs. 3) (Several of the mice in group 4 had bigger tumors, but others had smaller tumors, than mice in group 3). These results suggest that the expression of Ras^G12V^ in the cells has pushed the tumor-promoting potential to its outmost values (in group 3), and thus it could not have been promoted any further by CM^Ras-G12V+TNFα^ (in group 4).

A different pattern was revealed when metastasis was examined since highly pro-metastatic capacities were obtained by the Cells^Ras-G12V+TNFα^CM^Ras-G12V+TNFα^ group compared to all other treatment combinations. Here, a reliable criterion was tumor cell dissemination to LN adjacent to mammary fat pad (see “Methods”). Using the Maestro device in analyses of intact mice, we found that Cells^Ras-G12V+TNFα^CM^Ras-G12V+TNFα^ gave rise to significantly higher metastatic yield than each of the other three Cell-CM combinations. In mice inoculated by Cells^Ras-G12V+TNFα^CM^Ras-G12V+TNFα^, the lag period until dissemination of tumor cells to LN was shorter, and the percentage of mice with LN metastases was higher (83%) compared to all other Cell-CM combinations (12-36%; Figure 6C).

Of note was the fact that increased LN dissemination necessitated the expression of Ras^G12V^ in the cells as well as supplementation of CM derived from cells expressing hyper-activated Ras and stimulated by TNFα (=CM^RasG12V+TNFα^). Therefore, these results indicate that in order to metastasize, the cells required the expression of Ras^G12V^, but they also attest for the functional importance of the cooperativity between TNFα and Ras hyper-activation: Following joint activities of TNFα and Ras hyper-activation, the cells released high levels of tumor-promoting factors, which potentiated the metastatic potential of the tumor cells and their dissemination to LN.

## Discussion

The multi-factorial nature of malignant diseases has led researchers and clinicians to introduce novel therapeutic approaches based on combination therapy. Deciphering the molecular pathways involved in oncogenesis is essential for the development of personalized therapies, as is the identification of microenvironmental factors that induce intrinsic alterations in cells that undergo malignant transformation.

The findings presented in this study indicate that oncogenic events, such as hyper-activation of the Ras pathway, exacerbate the release of pro-malignancy chemokines (e.g., CXCL8 and CCL2) by MCF-7 human breast tumor cells. Moreover, these processes are further potentiated by inflammatory cytokines found in the tumor microenvironment, such as TNFα and IL-1β. The existence of such regulatory pathways is congruent with the significantly higher levels of TNFα, IL-1β, CXCL8 and CCL2 expression in breast tumors, as compared to normal breast cells [63], and with the ability of oncogenic Ras^G12V^ and TNFα (each alone) to up-regulate CXCL8 expression (through NF-κB activation) in tumor cells, as well as in other types of cells [33,34,36,64,65].

Our findings further demonstrate that TNFα transforms WT-Ras into a tumor-promoting entity. In that manner, the two components together induce the up-regulation of CXCL8 (and possibly of other tumor-promoting and angiogenic factors) and angiogenesis. Therefore, being highly expressed in breast tumors, TNFα may “bring the evil” out of WT-Ras and these two components together may lead to intensified pro-malignant effects that are deleterious in terms of angiogenesis and tumor progression. It is important to emphasize that following the activation of WT-Ras by TNFα, the cooperative activity between the activated form of WT-Ras and TNFα gives rise to CXCL8 up-regulation in a manner similar to that achieved by the constitutively active form of Ras^G12V^. Thus, the powerful ability of hyper-activated Ras + TNFα to promote metastasis (Figure 6) strongly suggests that TNFα activation of WT-Ras may lead to the dissemination of tumor cells.

The activation of WT-Ras by TNFα stimulation demonstrates that inflammatory factors can activate oncogenic pathways in breast tumor cells and promote disease progression in breast cancer. These findings are supported by several emerging studies in the field [40,41], and if evidence to such processes will be obtained by additional studies in breast cancer, they may have important therapeutic implications (please see below). From the mechanistic perspective, it is interesting to indicate that the TNFα-induced activation process of WT-Ras took hours to complete (Figure 3A), suggesting that TNFα induces the release of RTK ligands by the cells, which then activate the RTK-Ras pathway and lead (via NF-κB and AP-1) to increased transcription and protein expression of CXCL8. The involvement of RTK activation in this process is supported by published studies showing that TNFα induces the transactivation of ErbB2 in other cell systems (however, we note that those investigations did not directly address Ras activation or the effects of ErbB2-inducing activities on angiogenicity, tumor growth and metastasis [40,41]). Thus, in our system, it is possible that ErbB2 stimulation may be involved in the activation of WT-Ras by TNFα-induced signals. EGF may be one of the ligands that activate the ErbB2 pathway, as suggested by our finding that EGF induced CXCL8 expression in ErbB2-expressing cells (Figure 2A). It is possible that the release of EGF and many other RTK ligands (e.g., VEGF, bFGF, HGF) is induced as a consequence of TNFα activation, leading to RTK activation and then to cooperation in the release of CXCL8 by the tumor cells. Obviously, a comprehensive search based on protein arrays and neutralization assays would be required in order to identify the proteins that mediate the TNFα-induced WT-Ras activation observed in our system and such work would constitute an additional, full-scale research project. Nevertheless, the actual evidence for such TNFα activity significantly contributes to our understanding of the interactions between oncogenic events and microenvironmental processes in breast cancer.

Furthermore, in the malignant cells the hyper-activated Ras^G12V^ can act alone to promote the release of the angiogenic chemokines CXCL8 and CCL2. In contrast, in non-transformed cells, the induction of CXCL8 and CCL2 requires synergism between at least two oncogenic modifications: Ras^G12V^ and the down-regulation of p53. The latter pattern, evident in the non-transformed cells, is congruent with the regulatory patterns observed for other tumor-promoting characteristics in non-transformed cells [66]. In contrast, the transformed tumor cells already carried inherent alterations in their genetic/signaling setup. Thus, the silencing of p53 may have been replaced by modified activities of other protein/s in the tumor cells that exhibited a fully established malignancy phenotype. To identify candidate protein/s whose alteration may cooperate with Ras^G12V^, in-depth analyses of the genetic/signaling setup of the tumor cells would need to be carried out. That work would be appropriate for future studies, but is beyond the scope of the present investigation.

Our studies analyzing chemokine control by Ras^G12V^ ± p53 down-regulation have revealed similarities but also differences in the regulatory mechanisms determining the expression of CXCL8 and CCL2. As indicated above, Ras^G12V^ alone induced the release of CXCL8 and of CCL2. However, unlike CXCL8, CCL2 expression was reduced when p53 was down-regulated in the context of Ras hyper-activation. These findings agree with those of recent studies showing that p53 was bound to CCL2 5′UTR and that the knockdown of human p53 has led to strong negative regulation of CCL2 in macrophages [67,68]. Therefore, combining Ras hyper-activation with down-regulation of p53 demonstrated the existence of different regulatory circuits for CXCL8 as compared to CCL2.

Despite its ability to act alone in the tumor cells, Ras^G12V^ had a relatively minor effect on pro-malignancy activities in MCF-7 breast tumor cells (measured indirectly in terms of CXCL8 release), as compared to the inflammatory cytokines (Figure 1B). Actually, it was the joint activity of activated Ras and the inflammatory cytokines that had the most powerful effects on CXCL8 release and metastasis. Our seminal finding in this respect is that activities similar to those of Ras^G12V^ were achieved using WT-Ras following its activation by TNFα (Figure 2C). The strong metastasizing activities resulting out of the cooperation between hyper-activated Ras and TNFα suggest that the activation of WT-Ras by TNFα may give rise to more aggressive disease in breast cancer patients expressing WT-Ras and high levels of TNFα.

## Conclusions

In this study we have shown that TNFα rescued the tumor-promoting potential of WT-Ras and have demonstrated cooperativity between TNFα and activated Ras in metastasis. The mechanisms revealed in this study and in other supporting investigations suggest that oncogenic events are promoted by inflammatory signals that reside at the tumor microenvironment of breast tumors. Additional research in other breast tumor systems should be taken in order to substantiate these mechanisms, as they may have a significant impact on therapeutic approaches for the treatment of cases of breast cancer in which the tumors express high levels of TNFα and Ras is generally not mutated. In light of such mechanisms, we may need to consider the use of inhibitors of mutated (i.e., hyper-activated) Ras in patients who do not have any apparent constitutive activation of the oncogene due to its mutation and also express high levels of TNFα, as is the case for many breast cancer patients. Such inhibitors may include the farnesyl transferase inhibitors that are currently in clinical trials [2]. Furthermore, the interaction observed between TNFα and WT-Ras suggests that the therapeutic potential of Ras inhibitors would be enhanced if they were to be used together with the clinically available TNFα inhibitors, which have already been investigated in the context of several other types of malignancies and have proven to be safe [6]. Thus, the novel findings presented in our study have great clinical relevance, as they emphasize the need to consider the use of new therapeutic approaches in the treatment of breast cancer.

## Abbreviations

CAM: Chorioallantoic membrane; CM: Conditioned medium; DMSO: Dimethyl sulfoxide; EGF: Epidermal growth factor; FCS: Fetal calf serum; HRP: Horseredish peroxidase; IL-1β: Interleukin 1β; LN: Lymph nodes; RTK: Receptor Tyrosine Kinases; qRT-PCR: Quantitative real-time polymerase chain reaction; RBD: Ras binding domain; TNFα: Tumor necrosis factor α; WB: Western blot; WT: Wild-type.

## Competing interests

The authors declare that they have no competing interests.

## Authors’ contributions

TLR was the major contributor to the acquisition of data. She has made all of the experiments included in the study, and was involved in study design and conception. YL participated in many of the ELISA and WB analyses and in the animal model systems as well. TM is a research assistant who has produced the vectors used in the study. AA and MW contributed to tests using CAM. DB helped in ELISA assays determining the impact of the cytokines on chemokine release. HS was involved in the initial stages of study design, and provided Ras-expressing cells. VR participated in the design of the study. ABB was the principal investigator responsible for the whole study, including all its parts. All authors have approved the submission of the manuscript.

## Pre-publication history

The pre-publication history for this paper can be accessed here:

http://www.biomedcentral.com/1471-2407/14/158/prepub

## Supplementary Material

Additional file 1 **Validation of efficiencies of p53**^**shRNA **^**or GFP-RasG12V transfections. (A)** MCF-7 cells were stably transfected to express p53^shRNA^ or control vector. p53 levels were determined by WB. **(B)** MCF-7 cells were transiently transfected to express GFP-RasG12V or GFP-control vector. Transfection efficiencies were determined by flow cytometry of GFP-expressing cells. The activities of the Ras containing vectors in the transfected cells were verified by Erk activation (data not shown), and by quantitation of GTP-bound Ras levels, using RBD pull-down assays as shown in Figure 3A of manuscript. Click here for file

Additional file 2 **RasG12V induces the expression of CCL2 independently of deregulated p53.** MCF-7 cells were transfected to express p53^shRNA^, RasG12V, RasG12V+p53^shRNA^ or the appropriate control vectors. CCL2 levels were determined at the protein level in cell supernatants by ELISA **(A)**, and at the mRNA levels by qRT-PCR **(B)**. **p<0.01, ***p<0.001 compared to control cells. NS = Not significant. In both panels, a representative experiment of n≥3 is presented. Click here for file

Additional file 3 **ErbB2 and WT-Ras transfection yields, and Ras-related parameters in cells transfected by RasG12V and by WT-Ras. (A)** MCF-7 cells were transiently transfected to express ErbB2 or control vector. ErbB2 transfection efficiency was determined by qRT-PCR. ***p<0.001 for differences between ErbB2-transfected, and control vector-transfected cells. **(B)** MCF-7 cells were transiently transfected to express GFP-WT-Ras or GFP-control vector. Transfection efficiencies were determined by flow cytometry of GFP-expressing cells. The activities of the Ras containing vectors in the transfected cells were verified by EGF stimulation followed by quantitation of GTP-bound Ras levels, using RBD pull-down assays as shown in Figure 3A of manuscript. **(C)** Determination of GTP-bound Ras levels. The Figure shows the same WB results after brief film exposure and after longer film exposure, in order to demonstrate that the lower band (presumably translationally modified Ras) is expressed in WT-Ras-expressing cells, albeit in much lower levels than in RasG12V-expressing cells. General transfection yields of RasG12V were shown in Additional file 1B, and of WT-Ras in part B of the current Figure. **(D)** The figure shows the relatively low (and unstable) expression level of GTP-bound endogenous Ras (21 kDa) compared to over-expressed GFP-tagged GTP-bound WT-Ras (48 kDa) obtained following RBD assays (the results are from two different experiments: Exp. 1 - From non-stimulated tumor cells; Exp. 2 - From cells stimulated by TNFα for 7 minutes, which are conditions in which Ras is not activated (see Figure 3A). Click here for file

Additional file 4 **Validating the inhibitory functions of PD98059 on MAPK activation, indicated by levels of phosphorylated Erk.** MCF-7 cells were transiently transfected to express WT-Ras and were not-stimulated or stimulated by TNFα (50 ng/ml). This procedure was performed in the absence or presence of the MEK inhibitor PD98059 (50 μM), or its solubilizer (DMSO, at similar dilution). PD98059 was added to cell cultures 2 hr prior to stimulation of the cells by TNFα, and was present in culture throughout the duration of stimulation. Erk activation was determined by WB. Click here for file

Additional file 5 **IκBα levels in TNFα-stimulated WT-Ras expressing cells, and p65 down-regulation by shRNAs to p65. (A)** WT-Ras expressing MCF-7 cells were not-stimulated or stimulated by TNFα (50 ng/ml). Activation of the NF-κB pathway was analyzed by reduced levels of IκBα (=NF-κB inhibitor), determined by WB. A representative experiment of n=3 is presented. **(B)** Validation of the p65-reducing activities of siRNAs to p65, determined by WB (Inhibition levels: 42% and 62% inhibition for 25 nM and 35 nM siRNA to p65, respectively). Reduction of p65 expression by siRNA targeting p65 was denoted in n=3. Click here for file
