# Supplementary material for: The inflammatory cytokine TNFα cooperates with Ras in elevating metastasis and turns WT-Ras to a tumor-promoting entity in MCF-7 cells
Source: BMC Cancer. 2014 Mar 6;14:158. doi: 10.1186/1471-2407-14-158 (PMC4015419; doi:10.1186/1471-2407-14-158)
Supplement: Additional file 1 — Validation of efficiencies of p53shRNA or GFP-RasG12V transfections. (A) MCF-7 cells were stably transfected to express p53shRNA or control vector. p53 levels were determined by WB. (B) MCF-7 cells were transiently transfected to express GFP-RasG12V or GFP-control vector. Transfection efficiencies were determined by flow cytometry of GFP-expressing cells. The activities of the Ras containing vectors in the transfected cells were verified by Erk activation (data not shown), and by quantitation of GTP-bound Ras levels, using RBD pull-down assays as shown in Figure 3A of manuscript. [file 1471-2407-14-158-S1.pptx]

## Slide 1
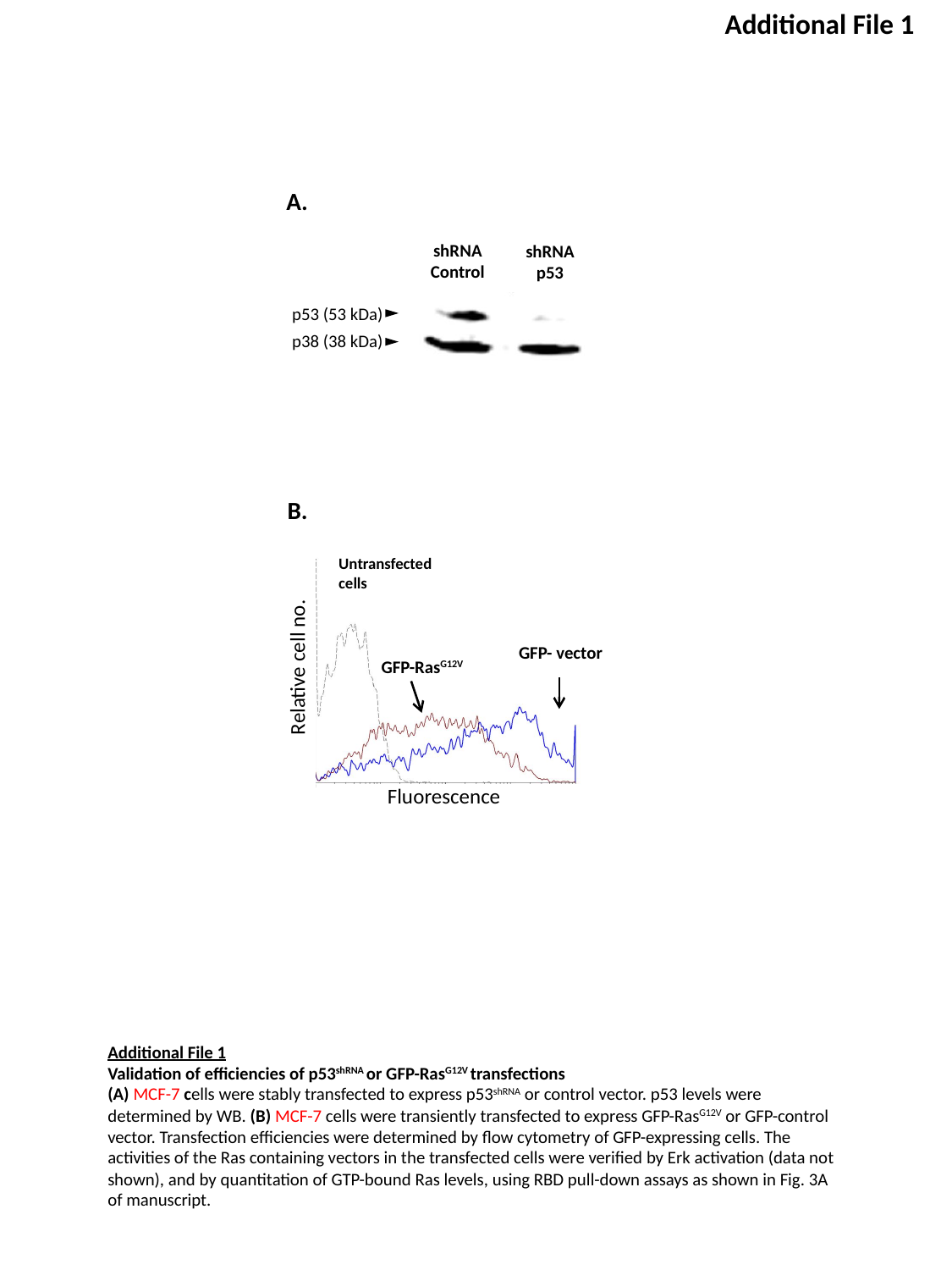

Additional File 1
A.
shRNA
Control
shRNA
p53
p53 (53 kDa)
p38 (38 kDa)
B.
Untransfected
cells
GFP- vector
Relative cell no.
GFP-RasG12V
Fluorescence
Additional File 1
Validation of efficiencies of p53shRNA or GFP-RasG12V transfections
(A) MCF-7 cells were stably transfected to express p53shRNA or control vector. p53 levels were determined by WB. (B) MCF-7 cells were transiently transfected to express GFP-RasG12V or GFP-control vector. Transfection efficiencies were determined by flow cytometry of GFP-expressing cells. The activities of the Ras containing vectors in the transfected cells were verified by Erk activation (data not shown), and by quantitation of GTP-bound Ras levels, using RBD pull-down assays as shown in Fig. 3A of manuscript.
