# Supplementary material for: The inflammatory cytokine TNFα cooperates with Ras in elevating metastasis and turns WT-Ras to a tumor-promoting entity in MCF-7 cells
Source: BMC Cancer. 2014 Mar 6;14:158. doi: 10.1186/1471-2407-14-158 (PMC4015419; doi:10.1186/1471-2407-14-158)
Supplement: Additional file 2 — RasG12V induces the expression of CCL2 independently of deregulated p53. MCF-7 cells were transfected to express p53shRNA, RasG12V, RasG12V+p53shRNA or the appropriate control vectors. CCL2 levels were determined at the protein level in cell supernatants by ELISA (A), and at the mRNA levels by qRT-PCR (B). **p<0.01, ***p<0.001 compared to control cells. NS = Not significant. In both panels, a representative experiment of n≥3 is presented. [file 1471-2407-14-158-S2.pptx]

## Slide 1
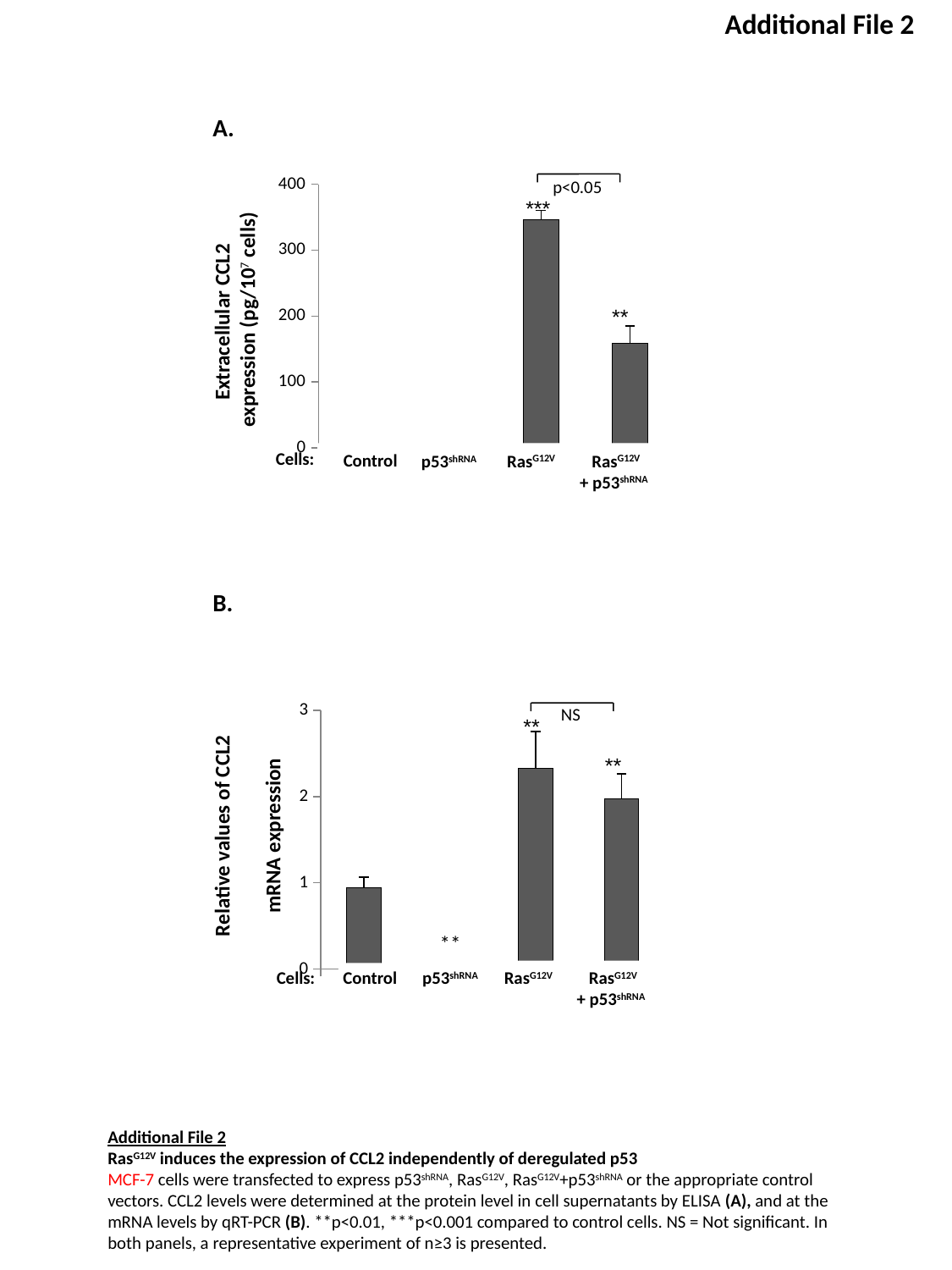

Additional File 2
A.
### Chart
| Category | |
|---|---|
| gfp | 0.0 |
| p53i | 0.0 |
| Ras control | 346.0 |
| x2 control | 158.0 |p<0.05
***
Extracellular CCL2
 expression (pg/107 cells)
**
Cells:
Control
RasG12V
RasG12V
+ p53shRNA
p53shRNA
B.
### Chart
| Category | |
|---|---|
| control | 0.9406666666666665 |
| p53i | 0.07326892109500811 |
| Ras G12V | 2.325301204819277 |
| Ras G12V + P53i | 1.9772565742715462 |NS
**
**
Relative values of CCL2
mRNA expression
***
Control
p53shRNA
RasG12V
RasG12V
+ p53shRNA
Cells:
Additional File 2
RasG12V induces the expression of CCL2 independently of deregulated p53
MCF-7 cells were transfected to express p53shRNA, RasG12V, RasG12V+p53shRNA or the appropriate control vectors. CCL2 levels were determined at the protein level in cell supernatants by ELISA (A), and at the mRNA levels by qRT-PCR (B). **p<0.01, ***p<0.001 compared to control cells. NS = Not significant. In both panels, a representative experiment of n≥3 is presented.
