# Supplementary material for: The inflammatory cytokine TNFα cooperates with Ras in elevating metastasis and turns WT-Ras to a tumor-promoting entity in MCF-7 cells
Source: BMC Cancer. 2014 Mar 6;14:158. doi: 10.1186/1471-2407-14-158 (PMC4015419; doi:10.1186/1471-2407-14-158)
Supplement: Additional file 3 — ErbB2 and WT-Ras transfection yields, and Ras-related parameters in cells transfected by RasG12V and by WT-Ras. (A) MCF-7 cells were transiently transfected to express ErbB2 or control vector. ErbB2 transfection efficiency was determined by qRT-PCR. ***p<0.001 for differences between ErbB2-transfected, and control vector-transfected cells. (B) MCF-7 cells were transiently transfected to express GFP-WT-Ras or GFP-control vector. Transfection efficiencies were determined by flow cytometry of GFP-expressing cells. The activities of the Ras containing vectors in the transfected cells were verified by EGF stimulation followed by quantitation of GTP-bound Ras levels, using RBD pull-down assays as shown in Figure 3A of manuscript. (C) Determination of GTP-bound Ras levels. The Figure shows the same WB results after brief film exposure and after longer film exposure, in order to demonstrate that the lower band (presumably translationally modified Ras) is expressed in WT-Ras-expressing cells, albeit in much lower levels than in RasG12V-expressing cells. General transfection yields of RasG12V were shown in Additional file 1B, and of WT-Ras in part B of the current Figure. (D) The figure shows the relatively low (and unstable) expression level of GTP-bound endogenous Ras (21 kDa) compared to over-expressed GFP-tagged GTP-bound WT-Ras (48 kDa) obtained following RBD assays (the results are from two different experiments: Exp. 1 - From non-stimulated tumor cells; Exp. 2 - From cells stimulated by TNFα for 7 minutes, which are conditions in which Ras is not activated (see Figure 3A). [file 1471-2407-14-158-S3.pptx]

## Slide 1
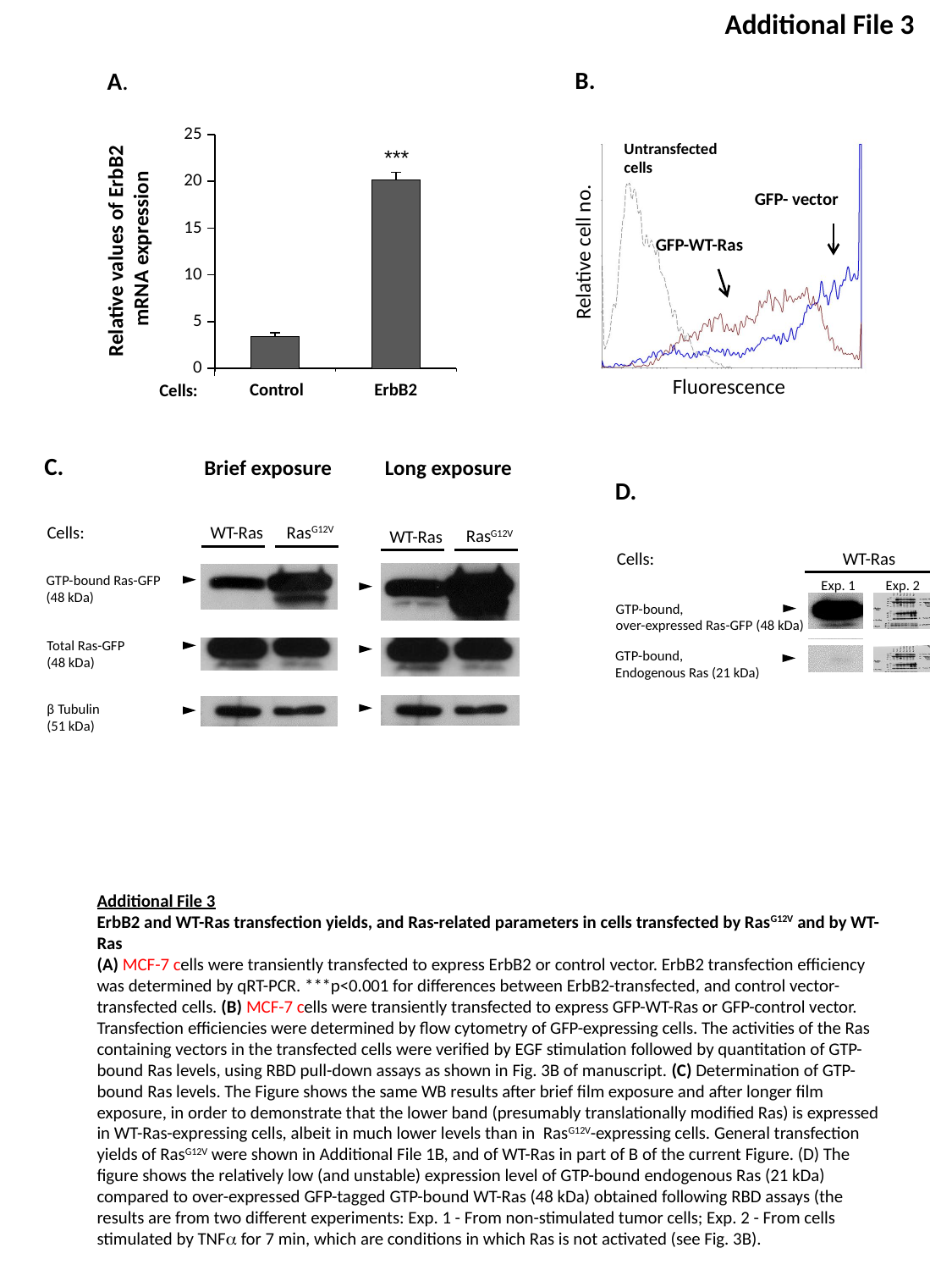

Additional File 3
B.
Untransfected
cells
GFP- vector
GFP-WT-Ras
Relative cell no.
A.
### Chart
| Category | |
|---|---|***
Relative values of ErbB2
mRNA expression
Control
ErbB2
Cells:
Fluorescence
C. 	 Brief exposure Long exposure
D.
RasG12V
WT-Ras
Cells:
GTP-bound Ras-GFP
(48 kDa)
Total Ras-GFP
(48 kDa)
β Tubulin
(51 kDa)
RasG12V
WT-Ras
Cells:
WT-Ras
Exp. 1
Exp. 2
GTP-bound,
over-expressed Ras-GFP (48 kDa)
GTP-bound,
Endogenous Ras (21 kDa)
Additional File 3
ErbB2 and WT-Ras transfection yields, and Ras-related parameters in cells transfected by RasG12V and by WT-Ras
(A) MCF-7 cells were transiently transfected to express ErbB2 or control vector. ErbB2 transfection efficiency was determined by qRT-PCR. ***p<0.001 for differences between ErbB2-transfected, and control vector-transfected cells. (B) MCF-7 cells were transiently transfected to express GFP-WT-Ras or GFP-control vector. Transfection efficiencies were determined by flow cytometry of GFP-expressing cells. The activities of the Ras containing vectors in the transfected cells were verified by EGF stimulation followed by quantitation of GTP-bound Ras levels, using RBD pull-down assays as shown in Fig. 3B of manuscript. (C) Determination of GTP-bound Ras levels. The Figure shows the same WB results after brief film exposure and after longer film exposure, in order to demonstrate that the lower band (presumably translationally modified Ras) is expressed in WT-Ras-expressing cells, albeit in much lower levels than in RasG12V-expressing cells. General transfection yields of RasG12V were shown in Additional File 1B, and of WT-Ras in part of B of the current Figure. (D) The figure shows the relatively low (and unstable) expression level of GTP-bound endogenous Ras (21 kDa) compared to over-expressed GFP-tagged GTP-bound WT-Ras (48 kDa) obtained following RBD assays (the results are from two different experiments: Exp. 1 - From non-stimulated tumor cells; Exp. 2 - From cells stimulated by TNFα for 7 min, which are conditions in which Ras is not activated (see Fig. 3B).
