# Supplementary material for: The inflammatory cytokine TNFα cooperates with Ras in elevating metastasis and turns WT-Ras to a tumor-promoting entity in MCF-7 cells
Source: BMC Cancer. 2014 Mar 6;14:158. doi: 10.1186/1471-2407-14-158 (PMC4015419; doi:10.1186/1471-2407-14-158)
Supplement: Additional file 4 — Validating the inhibitory functions of PD98059 on MAPK activation, indicated by levels of phosphorylated Erk. MCF-7 cells were transiently transfected to express WT-Ras and were not-stimulated or stimulated by TNFα (50 ng/ml). This procedure was performed in the absence or presence of the MEK inhibitor PD98059 (50 μM), or its solubilizer (DMSO, at similar dilution). PD98059 was added to cell cultures 2 hr prior to stimulation of the cells by TNFα, and was present in culture throughout the duration of stimulation. Erk activation was determined by WB. [file 1471-2407-14-158-S4.pptx]

## Slide 1
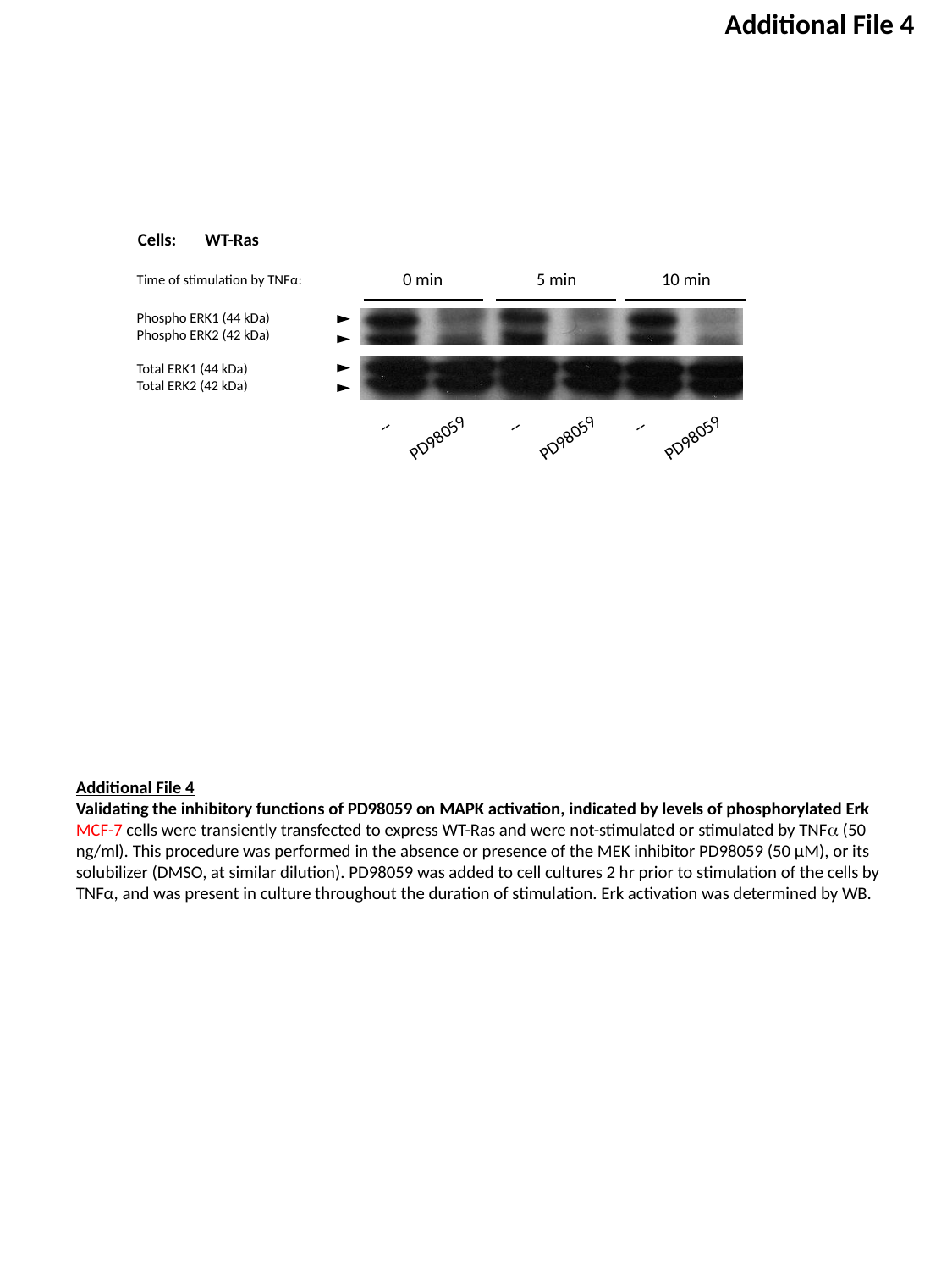

Additional File 4
Cells:
WT-Ras
0 min
5 min
10 min
Time of stimulation by TNFα:
Phospho ERK1 (44 kDa)
Phospho ERK2 (42 kDa)
Total ERK1 (44 kDa)
Total ERK2 (42 kDa)
--
--
--
PD98059
PD98059
PD98059
Additional File 4
Validating the inhibitory functions of PD98059 on MAPK activation, indicated by levels of phosphorylated Erk
MCF-7 cells were transiently transfected to express WT-Ras and were not-stimulated or stimulated by TNFα (50 ng/ml). This procedure was performed in the absence or presence of the MEK inhibitor PD98059 (50 μM), or its solubilizer (DMSO, at similar dilution). PD98059 was added to cell cultures 2 hr prior to stimulation of the cells by TNFα, and was present in culture throughout the duration of stimulation. Erk activation was determined by WB.
