# Supplementary material for: The inflammatory cytokine TNFα cooperates with Ras in elevating metastasis and turns WT-Ras to a tumor-promoting entity in MCF-7 cells
Source: BMC Cancer. 2014 Mar 6;14:158. doi: 10.1186/1471-2407-14-158 (PMC4015419; doi:10.1186/1471-2407-14-158)
Supplement: Additional file 5 — IκBα levels in TNFα-stimulated WT-Ras expressing cells, and p65 down-regulation by shRNAs to p65. (A) WT-Ras expressing MCF-7 cells were not-stimulated or stimulated by TNFα (50 ng/ml). Activation of the NF-κB pathway was analyzed by reduced levels of IκBα (=NF-κB inhibitor), determined by WB. A representative experiment of n=3 is presented. (B) Validation of the p65-reducing activities of siRNAs to p65, determined by WB (Inhibition levels: 42% and 62% inhibition for 25 nM and 35 nM siRNA to p65, respectively). Reduction of p65 expression by siRNA targeting p65 was denoted in n=3. [file 1471-2407-14-158-S5.pptx]

## Slide 1
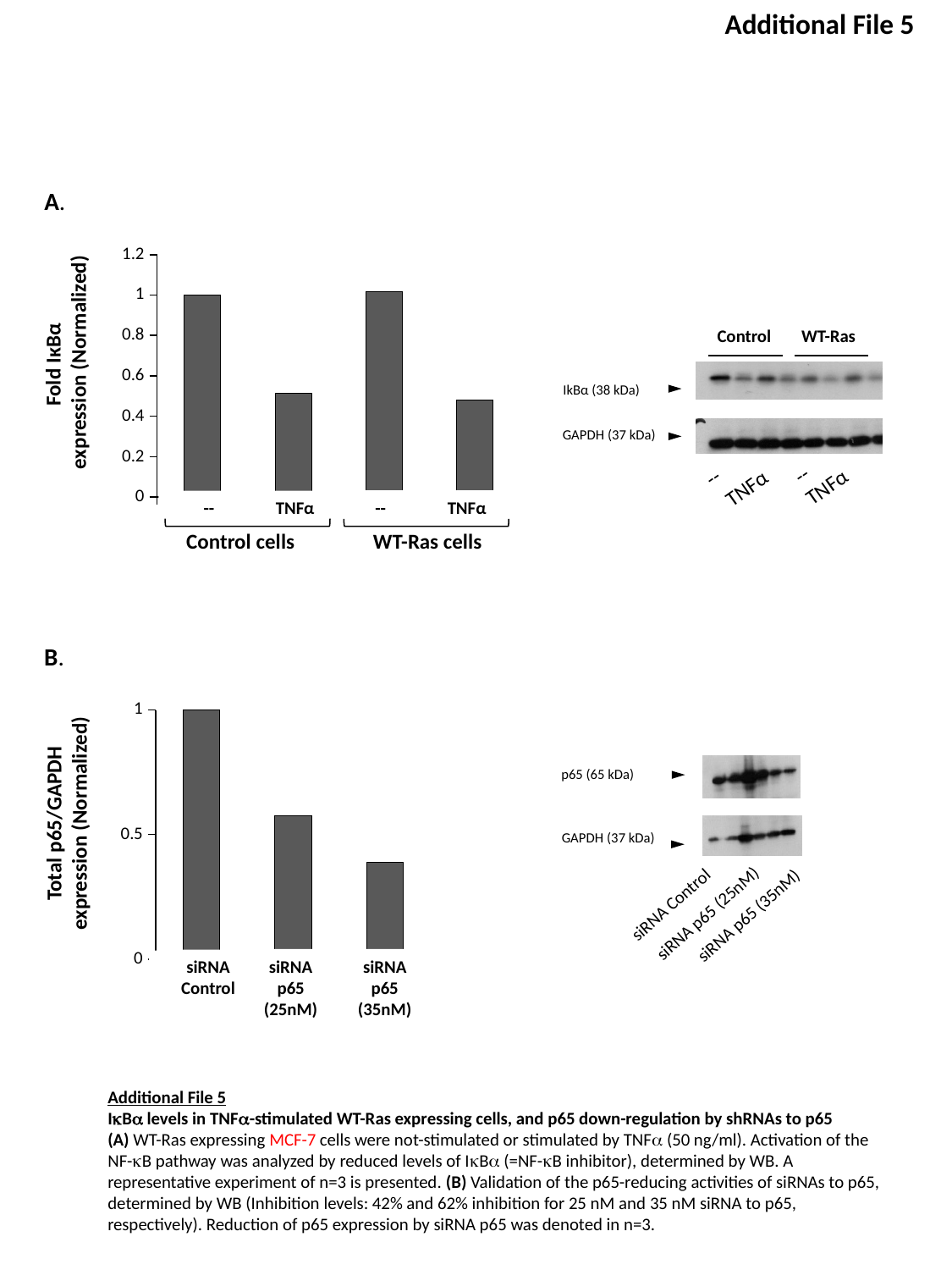

Additional File 5
A.
### Chart
| Category | |
|---|---|
| gfp - | 1.0 |
| gfp tnf | 0.5127256132795457 |
| ras - | 1.0173924557349177 |
| ras tnf | 0.4802460249261356 |
 Control
 WT-Ras
--
--
TNFα
TNFα
Fold IκBα
expression (Normalized)
IkBα (38 kDa)
GAPDH (37 kDa)
--
TNFα
TNFα
--
Control cells
WT-Ras cells
B.
### Chart
| Category | |
|---|---|
| Ras si all star | 1.0 |
| Ras si RelA 25nM | 0.5754839361681992 |
| Ras si RelA 35nM | 0.3885718305175208 |
p65 (65 kDa)
Total p65/GAPDH expression (Normalized)
GAPDH (37 kDa)
siRNA p65 (25nM)
siRNA p65 (35nM)
 siRNA Control
siRNA
Control
siRNA
p65 (25nM)
siRNA
p65 (35nM)
Additional File 5
IκBα levels in TNFα-stimulated WT-Ras expressing cells, and p65 down-regulation by shRNAs to p65
(A) WT-Ras expressing MCF-7 cells were not-stimulated or stimulated by TNFα (50 ng/ml). Activation of the NF-κB pathway was analyzed by reduced levels of IκBα (=NF-κB inhibitor), determined by WB. A representative experiment of n=3 is presented. (B) Validation of the p65-reducing activities of siRNAs to p65, determined by WB (Inhibition levels: 42% and 62% inhibition for 25 nM and 35 nM siRNA to p65, respectively). Reduction of p65 expression by siRNA p65 was denoted in n=3.
